# A comparison between anorganic bone and collagen-preserving bone xenografts for alveolar ridge preservation: systematic review and future perspectives

**DOI:** 10.1186/s40902-022-00349-3

**Published:** 2022-07-12

**Authors:** Danilo Alessio Di Stefano, Francesco Orlando, Marco Ottobelli, Davide Fiori, Umberto Garagiola

**Affiliations:** 1 Private Practice, Centro Odontoiatrico e Protesico Civitali S.R.L., Milan, Italy; 2 Department of Dentistry, Vita-Salute San Raffaele University Milan, Milan, Italy; 3 Dental School, Vita-Salute University IRCCS San Raffaele, Milan, Italy; 4 Department of Biomedical, Surgical and Dental Sciences, University of Milan, Milan, Italy

**Keywords:** Alveolar ridge preservation, Alveolar socket, Bone xenograft, Anorganic bone, Collagen-preserving bone

## Abstract

After tooth extraction, dimensional changes affect the alveolar socket, leading to loss in alveolar bone height and width. Histological modifications also occur, with initial formation of a blood clot that is replaced with granulation tissue and subsequently with a provisional connective tissue matrix. Spontaneous healing ends with socket filling with woven bone, which is gradually replaced with lamellar bone and bone marrow. Adequate alveolar ridge dimensions and bone quality are required to assure optimal stability and osseointegration following dental implant placement. When a tooth is extracted, alveolar ridge preservation (ARP) procedures are an effective method to prevent collapse of the post-extraction socket. Heterologous bone is widely chosen by clinicians for ARP, and anorganic bone xenografts (ABXs) made bioinert by heat treatment represents the most used biomaterial in clinical applications. Collagen-preserving bone xenografts (CBXs) made of porcine or equine bone are fabricated by less invasive chemical or enzymatic treatments to remove xenogenic antigens, and these are also effective in preserving post-extraction sites. Clinical differences between anorganic bone substitutes and collagen-preserving materials are not well documented in the literature but understanding these differences could clarify how processing protocols influence biomaterial behavior in situ. This systematic review of the literature compares the dimensional changes and histological features of ABXs versus CBXs in ridge preservation procedures to promote awareness of different bone xenograft efficacies in stimulating the healing of post-extraction sockets.

## Introduction

Bone grafts and substitutes are increasingly used in dental implantology due to the growing need for replacing insufficient alveolar bone before implant placement [1]. One of the primary reasons for bone deficiency is tooth loss due to periodontal disease, tooth fracture/trauma, periapical lesions, or other pathological conditions [2]. Experimental evidence collected through animal [3, 4] and human [5, 6] studies demonstrated that after tooth extraction, the alveolar bone undergoes a remodeling process with consequent resorption of the vestibular cortical bone and gradual loosening of the marrow component of the alveolus. Bone reduction is mainly due to the lack of intraosseous stimulation normally provided by periodontal ligament fibers [1], and it is probably correlated with disruption of the blood supply and osteoclastic activity that occur after tooth extraction [7, 8]. The greatest amount of alveolar socket resorption occurs in the first 3 months after extraction, with a 30% reduction of the alveolar ridge (3.87 mm in width and 1.67 mm in height) [9–11]. Dimensional changes take place up to 1 year thereafter, with about 50% total reduction (5–7 mm in width) of the alveolar ridge within 12 months post-extraction [8, 12, 13]. Interestingly, alveolar ridge resorption is more severe on the buccal side than on the lingual side [3, 11].

Bone dimensional changes at the post-extraction site influence the subsequent implant treatment plan; this important clinical issue is currently treated by alveolar ridge preservation (ARP) techniques. Also known as “socket preservation”, ARP includes methods of counteracting alveolar bone resorption after tooth extraction by (1) maintaining the soft and hard ridge components, (2) sustaining bone regeneration within the socket, and (3) facilitating prosthetically driven implant placement [10, 14–16]. Recent systematic reviews with meta-analyses demonstrated that in comparison with unassisted socket healing, ARP procedures reduce alveolar bone dimensional changes and can promote bone regeneration at the post-extraction site [17–20]. Furthermore, dental implants inserted into ARP-treated sites exhibited a high survival rate [20]. ARP is most commonly achieved by filling the alveolar socket with a bone grafting material immediately after tooth extraction [13]. The ideal properties of bone substitute materials include osteogenic, osteoinductive, and osteoconductive capacities similar to the native bone, as well as high biocompatibility and low immunogenicity [21]. Materials currently being investigated for ARP use include autologous bone, demineralized or mineralized freeze-dried bone allografts, xenogenic bone, alloplastic polymers, bioactive glasses, and composite ceramic substitutes [22, 23]. Among these options, xenografts seem to avoid comorbidity issues, ensuring larger availability from animal rather than human bone and avoiding tissue-banking costs. Furthermore, xenogenic bone shows better resorption and integration capacity with the host tissue than synthetic materials.

Amongst heterologous materials, the use of anorganic bone xenografts (ABXs) for ARP procedures is well supported by scientific literature, with successful outcomes obtained in both animal preclinical studies and human randomized clinical trials [24–26]. ABXs are produced by exposure to heat and chemical extraction processes to remove immunogenic and organic components and are then prepared as porous grains (0.25–2 mm) [25, 27]. Regardless of the species of origin (i.e., bovine or porcine), ABXs exhibit structures and properties similar to their human counterparts, with clinical evidence demonstrating comparable outcomes among xenografts from different sources [28]. Besides demonstrating good osteoconductive properties, heat-treated ABXs also have poor resorption rates [29–31].

Another xenogenic biomaterial successfully used for ARP procedures is non-heat treated cortico-cancellous porcine bone (CPB), which is subjected to a collagen-preserving chemical process for immunogenic component removal and is then prepared as micro-porous particles (diameter 0.6–1 mm) [32]. These collagen-containing porcine bone grafts possess excellent osteoconductive properties and do not cause inflammatory infiltration [33, 34]. These biomaterials also show clear signs of resorption/remodeling after socket grafting, with the formation of scalloped lacunae [35, 36].

Successful ARP outcomes were recently achieved by grafting the post-extraction socket with enzyme-deantigenic equine bone (EDEB), which also consists of a mixture of cancellous and cortical bone granules (diameter 0.25–1 mm) made non-antigenic with digestive enzymes [37, 38]. In addition to ARP procedures, EDEB was used with satisfactory results in peri-apical cyst-removal management [39], horizontal/vertical ridge and sinus augmentation [40–42], and orthopedic applications [43–45].

Unlike ABXs, CPB and EDEB are collagen-preserving bone xenografts (CBXs) manufactured by chemical (CPB) or enzymatic (EDEB) treatment that maintains type I bone collagen in its native state. This may offer important advantages in terms of stimulation of the regenerative process, integration with the host tissue, and graft resorption rate [38, 46, 47].

There is scant evidence in the literature about which of these two classes of xenogenic bone substitutes—ABXs or CBXs—is better for preserving post-extraction sockets. To the best of our knowledge, only three clinical trials have compared the dimensional and histomorphometric outcomes of ABXs and CBXs, with one suggesting that CBX might produce a better healing pattern, and one demonstrating that collagen-preserving material obtained by enzymatic treatment ensures better bone regeneration and graft resorption [31, 36, 38]. This systematic review was performed to (1) compare bone dimensional changes after tooth extraction and ARP by ABXs or CBXs and (2) analyze and compare histologic and histomorphometric outcomes for post-extraction sites grafted with the two types of bone substitutes.

## Materials and methods

The present review was designed and conducted according to PRISMA (Preferred Reporting Items Systematic review and Meta-Analyses) guidelines [48, 49].

### Focused questions

1. Bone dimensional changes: which bone xenograft between ABXs and CBXs best preserves the horizontal and vertical ridge dimensions after ARP?
2. Bone regeneration: which bone xenograft between ABXs and CBXs achieves the best percentage of new bone formation after ARP?

### Eligibility criteria

The inclusion criteria of studies for this systematic review were organized according to the PICOT format [50].

Patients (P): Adult patients (age between 18 and 85 years) undergoing ARP procedures after tooth extraction.

Intervention (I): ARP strategies based on the use of anorganic bone or CBXs to fill the alveolar socket.

Comparison (C): All grafting procedures were considered for comparison, including different xenograft or allograft/synthetic materials, the use of a barrier membrane alone or in combination with the graft, and the non-intervention strategy (i.e., spontaneous healing).

Outcomes (O): The primary outcomes included: (1) bone dimensional changes evaluated by horizontal and vertical measurement of the alveolar ridge; (2) bone regeneration evaluated by histomorphometric analyses of bone biopsies to assess the percentage of newly formed/vital bone, as well as the amounts of connective tissue and residual grafting material. The secondary outcomes included: (1) change in buccal plate thickness; (2) bone volume alteration following extraction; (3) complications; (4) histological healing characteristics; (5) site eligibility for placement of an adequate size dental implant with or without further augmentation; (6) patient-reported outcomes.

Time (T): Follow-up after the surgical intervention at least 3 months.

Studies were filtered by considering only clinical trials investigating ABXs or CBXs for alveolar ridge preservation after tooth extraction. The exclusion criteria were the following: (1) cross-sectional studies, case series, case reports, pre-clinical studies, in vitro investigations; (2) studies reporting different primary outcome measures (i.e., soft tissue changes, implant stability after ARP); (3) clinical studies not clearly meeting the inclusion criteria.

### Search strategy

Electronic databases (MEDLINE (PubMed), EMBASE, Cochrane Central Register of Controlled Trials, and Scopus) were methodically searched for eligible articles by using the following combinations of keywords and MeSH terms: “alveolar ridge preservation”, “alveolar preservation”, “ridge preservation”, “socket preservation”, “post-extractive socket”, “bone xenograft”, “bovine bone xenograft”, “deproteinized bovine bone”, “deproteinized bovine bone matrix”, “deproteinized porcine bone”, “porcine bone xenograft”, “equine bone xenograft”, “animal bone graft”, “animal bone substitute”, “heterologous bone graft”, “heterologous bone substitute”. Only studies in English language were included, whereas no time restrictions were set to filter articles.

### Study selection

Titles and abstracts obtained by the electronic search were initially screened by the five authors. The full paper was considered for studies that had a missing or insufficient abstract to determine eligibility. Full-text versions of all the eligible articles were then obtained and carefully investigated by the five authors for final inclusion. The five authors performed parallel independent assessment and selection of the manuscripts and they had to agree on the inclusion/exclusion criteria and the finally included papers. Any disagreements among reviewers were resolved through discussion and consensus with the supervision by the corresponding author. At the end of the selection process, a total of 39 studies was included in the systematic review.

### Data collection

Included studies were analyzed by recording the following primary outcome measures:

1. Horizontal dimensional changes of the alveolar socket (in mm), measured clinically or radiographically at the level of the crest, or at different vertical distances from the crest or landmarks (i.e., adjacent teeth or implants).
2. Vertical dimensional changes of the alveolar socket (in mm) measured clinically or radiographically either at the level of the crest or at the buccal and palatal/lingual aspect.
3. Histomorphometric evaluation of the percentage of newly formed bone (NFB), soft tissues, residual graft particles.

Dimensional outcomes were calculated as differences between baseline (i.e., soon after tooth extraction) and the clinical/radiological situation at follow-up. Measures could be either positive or negative, with negative and positive values indicating a loss/reduction and gain/increase of ridge dimensions, respectively.

Collected data were summarized by preparing schematic tables regarding (1) main study characteristics (i.e., first author, year of publication, study design, patient characteristics, surgical interventions, type of bone xenograft, reported outcomes), (2) dimensional outcomes of ARP procedures using ABXs, (3) dimensional outcomes of ARP procedures using CBXs, (4) histomorphometric outcomes of ARP procedures using ABXs, and (5) histomorphometric outcomes of ARP procedures using CBXs.

Due to high variability of data and heterogeneity of the selected clinical trials, no meta-analysis could be performed to statistically compare the clinical outcomes of bone xenografts in ARP procedures.

### Risk of bias assessment

Quality evaluation on the selected studies was performed according to the Cochrane Handbook for Systematic Reviews of Interventions [51]. The following quality criteria were verified: random generation, allocation concealment, blinding of participants and personnel, blinding of outcome assessment, incomplete outcome data, and other sources of bias.

## Results

### Study selection

**Fig. 1:**
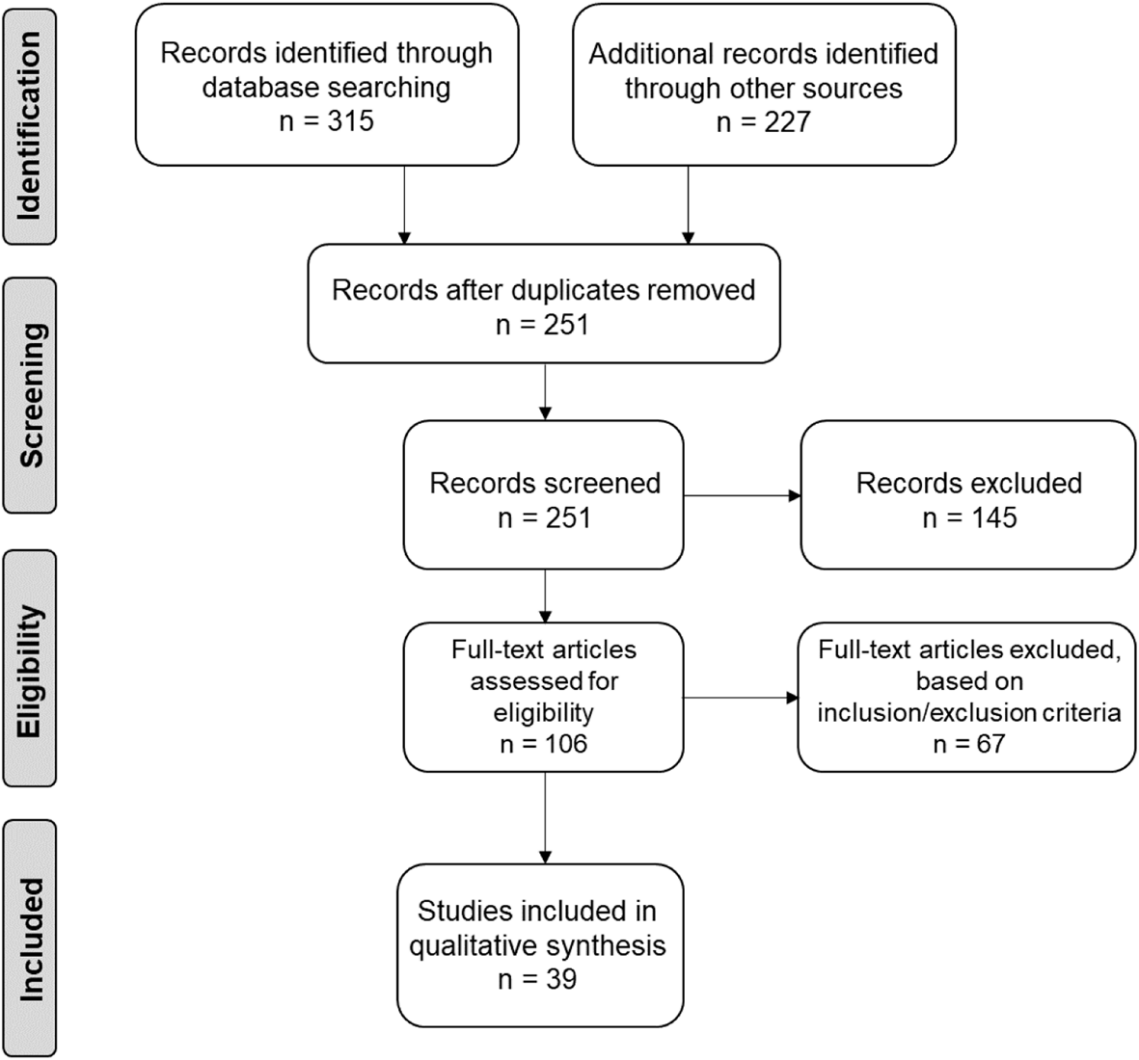
PRISMA flow diagram displaying the search results

The results of the literature search are shown in the PRISMA flow diagram (Fig. 1). The initial search yielded 542 total records. After removal of duplicates, 251 articles underwent title and abstract screening, which led to the exclusion of 145 records. Thus, 106 articles remained for full-text assessment (Fig. 1). There were 30 papers evaluating ARP techniques based on the use of ABXs [23, 26, 28, 52–78] and 9 papers evaluating ARP techniques based on the use of CBXs [31, 34, 36, 38, 79–83] that were eligible for inclusion (Table 1). Among these, 27 records about ABXs [23, 28, 53, 54, 56–78] and 7 records about CBXs [31, 34, 36, 79–81, 83] were eligible for inclusion in the analysis of horizontal and vertical changes of the alveolar ridge (Tables 2 and 3). In parallel, 19 records about ABXs [26, 28, 52–58, 60, 63, 66–68, 70, 73, 75–77] and 4 [31, 38, 79, 82] records about CBXs were eligible for inclusion in the analysis of histomorphometric outcomes (Tables 4 and 5). The most common reasons for exclusion were (1) not considering a xenograft material for ARP; (2) reporting of changes related to alveolar ridge volume, basal/superior surfaces, and shape; (3) reporting of implant primary and secondary stability as outcome variables; and (4) presenting case reports or case series with limited number of patients (*n* < 10).

**Table 1:** Characteristics of the studies included in the systematic review (n = 39)

**First Author(Publication Year)**	**Study design**	**Patient characteristics**	**Surgical interventions**	**Type of bone xenograft**	**Outcome variables**	
					**Primary outcomes**	**Secondary outcomes**
Carmagnola(2003) [52]	Prospective clinical trial	N = 21 (8F/13M) MEAN AGE: 56.5 ± 9.7 years AGE RANGE: 39–76 years Extraction sockets: 31	Tooth extraction ARP procedure Implant placement	ABX	- Histomorphometric measures	- Site eligibility for implant placement
Vance(2004) [53]	RCT	N = 24 (15F/9M) MEAN AGE: 56 ± 11 years Extraction sockets: 24	Tooth extraction ARP procedure Implant placement	ABX	- Ridge dimensional changes - Histomorphometric measures	- Changes in soft tissue thickness - Histological healing characteristics - Site eligibility for implant placement
Mardas(2010) [54]	RCT	N = 27 (21F/6M) MEAN AGE: 37.3 ± 11.4 years AGE RANGE: 20–58 years Extraction sockets: 26	Tooth extraction ARP procedure Implant placement	ABX	- Ridge dimensional changes - Histomorphometric measures	- Site eligibility for implant placement - Gingival recession - Probing pocket depth
Heberer(2011) [55]	Prospective clinical trial	N = 25 (10F/15M) MEAN AGE: 49.9 years AGE RANGE: 36–67 years Extraction sockets: 39	Tooth extraction ARP procedure Implant placement	Collagenated ABX	- Histomorphometric measures	- Histological healing characteristics - Site eligibility for implant placement - Complications
Nam (2011) [56]	Prospective clinical trial	N = 42 (22F/20M) AGE RANGE: 36–65 years Extraction sockets: 44	Tooth extraction ARP procedure Implant placement	ABX +/- coating with collagen-binding peptide	- Ridge dimensional changes - Histomorphometric measures	- Site eligibility for implant placement
Gholami (2012) [57]	RCT	N = 12 (8F/4M) MEAN AGE: 44.6 ± 11.4 years AGE RANGE: 21–60 years Extraction sockets: 28	Tooth extraction ARP procedure Implant placement	ABX	- Ridge dimensional changes - Histomorphometric measures	- Histological healing characteristics - Site eligibility for implant placement
Cook(2013) [58]	RCT	N = 44 (26F/18M) MEAN AGE: 56 years AGE RANGE: 23–78 years Extraction sockets: 40	Tooth extraction ARP procedure	Collagenated ABX	- Ridge dimensional changes - Histomorphometric measures	- Buccal plate thickness - Site eligibility for implant placement
Jung (2013) [59]	RCT	N = 40 (23F/17M) MEAN AGE: 55 years Extraction sockets: 40	Tooth extraction ARP procedure	Collagenated ABX	- Ridge dimensional changes	- Buccal plate thickness - Complications
Calasans-Maia (2014) [60]	RCT	N = 20 (13F/7M) AGE RANGE: 30–60 years Extraction sockets: 20	Tooth extraction ARP procedure Implant placement	ABX	- Ridge dimensional changes - Histomorphometric measures	- Site eligibility for implant placement - Complications
Cardaropoli (2014) [61]	RCT	N = 41 (17F/24M) MEAN AGE: 47.2 ± 12.9 years Extraction sockets: 48	Tooth extraction ARP procedure Implant placement	Collagenated ABX	- Ridge dimensional changes	- Buccal plate thickness - Complications
Pang (2014) [62]	Prospective, randomized clinical trial	N = 30 (16F/14M) MEAN AGE: 37 years AGE RANGE: 22–47 years Extraction sockets: 30	Tooth extraction ARP procedure Implant placement	ABX	- Ridge dimensional changes	- Bone volume changes - Complications
Milani (2016) [26]	Prospective, randomized clinical trial	N = 20 (16F/14M) MEAN AGE: 50.8 years Extraction sockets: 20	Tooth extraction ARP procedure Implant placement	ABX	- Histomorphometric measures	- Site eligibility for implant placement - Histological healing characteristics
Scheyer (2016) [63]	RCT	N = 40 AGE RANGE: 18–70 years Extraction sockets: 40	Tooth extraction ARP procedure Implant placement	Collagenated ABX	- Ridge dimensional changes - Histomorphometric measures	- Site eligibility for implant placement - Histological healing characteristics
Iorio-Siciliano (2017) [64]	RCT	N = 20 (9F/11M) MEAN AGE: 39.2 years Extraction sockets: 20	Tooth extraction ARP procedure Implant placement	Collagenated ABX	- Ridge dimensional changes	- Site eligibility for implant placement - Complications
Lim (2017) [65]	RCT	N = 30 (12F/18M) MEAN AGE: 50.2 ± 15.7 years AGE RANGE: 22–82 years Extraction sockets: 30	Tooth extraction ARP procedure Implant placement	Collagenated bovine or porcine ABX	- Ridge dimensional changes	- Site eligibility for implant placement - Complications
Nart (2017) [66]	RCT	N = 21 (15F/6M) MEAN AGE: 56.76 years Extraction sockets: 22	Tooth extraction ARP procedure Implant placement	ABX +/- heterologous collagen	- Ridge dimensional changes - Histomorphometric measures	- Buccal plate thickness - Site eligibility for implant placement - Histological healing characteristics - Complications
Pang (2017) [67]	RCT	N = 24 (13F/11M) MEDIAN AGE: 58 years Extraction sockets: 33	Tooth extraction ARP procedure Implant placement	ABX	- Ridge dimensional changes - Histomorphometric measures	- Site eligibility for implant placement - Implant stability - Complications
Serrano Mendez (2017) [68]	RCT	N = 20 (10F/10M) MEAN AGE: 44 years Extraction sockets: 20	Tooth extraction ARP procedure Implant placement	Collagenated ABX	- Ridge dimensional changes - Histomorphometric measures	- Site eligibility for implant placement - Histological healing characteristics
Fischer (2018) [69]	RCT	N = 40 (24F/16M) MEAN AGE: 55.7 ± 14.8 years AGE RANGE: 18–80 years Extraction sockets: 40	Tooth extraction ARP procedure Implant placement	ABX	- Ridge dimensional changes	- Site eligibility for implant placement - Need for bone augmentation
Shim (2018) [70]	RCT	N = 15 (3F/12M) AGE RANGE: 39–77 years Extraction sockets: 20	Tooth extraction ARP procedure Implant placement	ABX	- Ridge dimensional changes - Histomorphometric measures	- Histological healing characteristics - Complications
Tomasi (2018) [71]	RCT	N = 27 (16F/11M) MEAN AGE: 52 years AGE RANGE: 38–79 years Extraction sockets: 40	Tooth extraction ARP procedure Implant placement	Collagenated ABX	- Ridge dimensional changes	- Site eligibility for implant placement - Complications
Cha (2019) [72]	RCT	N = 39 (13F/26M) MEAN AGE: 53.4 years Extraction sockets: 39	Tooth extraction ARP procedure Implant placement	Collagenated ABX	- Ridge dimensional changes	- Site eligibility for implant placement - Need for bone augmentation - Complications
Lim (2019) [73]	RCT	N = 29 (8F/21M) MEAN AGE: 54.2 years Extraction sockets: 29	Tooth extraction ARP procedure Implant placement	Collagenated ABX	- Ridge dimensional changes - Histomorphometric measures	- Site eligibility for implant placement - Need for bone augmentation - Change of marginal bone level - Implant survival rate
Llanos (2019) [74]	RCT	N = 65 (31F/34M) MEAN AGE: 42.6 years Extraction sockets: 40	Tooth extraction ARP procedure Implant placement	ABX +/- heterlogous collagen	- Ridge dimensional changes	- Buccal plate thickness - Site eligibility for implant placement - Complications
Machtei (2019) [75]	RCT	N = 33 (12F/21M) MEAN AGE: 63.9 ± 8.1 years AGE RANGE: 45–80 years Extraction sockets: 33	Tooth extraction ARP procedure Implant placement	ABX	- Ridge dimensional changes - Histomorphometric measures	- Buccal plate thickness - Histological healing characteristics - Pain scores
Santana (2019) [76]	RCT	N = 32 (18F/14M) MEAN AGE: 42 ± 8 years AGE RANGE: 34–52 years Extraction sockets: 41	Tooth extraction ARP procedure Implant placement	ABX	- Ridge dimensional changes - Histomorphometric measures	- Complications
Taschieri (2019) [77]	Prospective clinical trial	N = 20 (8F/12M) MEAN AGE: 42.8 ± 5.1 years AGE RANGE: 33–50 years Extraction sockets: 20	Tooth extraction ARP procedure Implant placement	ABX	- Ridge dimensional changes - Histomorphometric measures	- Site eligibility for implant placement - Histological healing characteristics - Complications - Patients’ quality of life - Pain scores
Iorio-Siciliano (2020) [78]	RCT	N = 40 (22F/18M) MEAN AGE: 40.3 years Extraction sockets: 40	Tooth extraction ARP procedure Implant placement	ABX +/- heterologous collagen	- Ridge dimensional changes	- Site eligibility for implant placement
Lai (2020) [28]	RCT	N = 44 (27F/17M) MEAN AGE: 57 years AGE RANGE: 24–83 years Extraction sockets: 38	Tooth extraction ARP procedure Implant placement	Bovine or porcine ABX	- Ridge dimensional changes - Histomorphometric measures	- Buccal plate thickness - Site eligibility for implant placement - Implant stability - Histological healing characteristics - Complications
Lee (2020) [23]	RCT	N = 28 (10F/18M) MEAN AGE: 52.9 years AGE RANGE: 22–74 years Extraction sockets: 28	Tooth extraction ARP procedure Implant placement	Collagenated ABX +/- EMD	- Ridge dimensional changes	- Site eligibility for implant placement - Early postoperative discomfort - Soft tissue wound healing
Barone (2008) [79]	RCT	N = 40 (24F/16M) AGE RANGE: 26–69 years Extraction sockets: 40	Tooth extraction ARP procedure Implant placement	CBX	- Ridge dimensional changes - Histomorphometric measures	- Plaque index, gingival index and bleeding on probing - Site eligibility for implant placement - Histological healing characteristics - Complications
Barone (2013) [34]	Prospective randomized clinical trial	N = 59 (39F/20M) MEAN AGE: 40.5 years AGE RANGE: 20–63 years Extraction sockets: 58	Tooth extraction ARP procedure Implant placement	CBX	- Ridge dimensional changes	- Plaque index and gingival index - Site eligibility for implant placement - Need for bone augmentation - Length and diameter of implants
Festa (2013) [80]	RCT	N = 15 (9F/6M) MEAN AGE: 40.5 years AGE RANGE: 28–58 years Extraction sockets: 30	Tooth extraction ARP procedure Implant placement	CBX	- Ridge dimensional changes	- Probing pocket depth, gingival recession and bleeding on probing - Site eligibility for implant placement - Need for bone augmentation - Complications
Barone (2014) [81]	RCT	N = 64 (38F/26M) MEAN AGE: 32.7 ± 12.4 years AGE RANGE: 18–47 years Extraction sockets: 64	Tooth extraction ARP procedure Implant placement	CBX	- Ridge dimensional changes	- Site eligibility for implant placement - Need for bone augmentation - Complications
Barone (2015) [82]	RCT	N = 34 (20F/14M) AGE RANGE: 21–71 years Extraction sockets: 34	Tooth extraction ARP procedure Implant placement	CBX	- Histomorphometric measures	- Site eligibility for implant placement - Histological healing characteristics
Barone (2017) [31]	RCT	N = 90 (54F/36M) AGE RANGE: 25–70 years Extraction sockets: 90	Tooth extraction ARP procedure Implant placement	ABX *vs.* CBX	- Ridge dimensional changes - Histomorphometric measures	- Tooth site: premolar or molar - Buccal bone thickness
Marconcini (2018) [36]	RCT	N = 42 (25F/17M) MEAN AGE: 52.8 ± 2.31 years Extraction sockets: 42	Tooth extraction ARP procedure Implant placement	ABX *vs.* CBX	- Ridge dimensional changes	- Need for bone augmentation before implant placement - Esthetic outcome of the peri-implant mucosa - Implant success and survival rates - Complications
Di Stefano (2019) [38]	Retrospective clinical trial	N = 46 (21F/25M) MEAN AGE: 54 years AGE RANGE: 43–75 years Extraction sockets: 84	Tooth extraction ARP procedure Implant placement	ABX *vs.* CBX	- Histomorphometric measures	- Histological healing characteristics - Complications
Roberto (2021) [83]	Retrospective clinical trial	N = 54 (34F/20M) MEAN AGE: 53.8 ± 7.1 years AGE RANGE: 41.8–69.1 years Extraction sockets: 54	Tooth extraction ARP procedure Implant placement	CBX	- Ridge dimensional changes	- Long-term maintenance of buccal plate - Complications

Abbreviations: *ABX* anorganic bone xenograft, *ARP* alveolar ridge preservation, *CBX* collagen-preserving bone xenograft, *EMD* Enamel matrix derivative, *RCT* randomized controlled trial

**Table 2:** Dimensional changes of the alveolar ridge after ARP procedures with ABXs. Data are presented as Mean ± SD

Reference	Untreated group	Treated group 1 (ARP)	Treated group 2 (ARP)	Treated group 3 (ARP)	Description of the endpoint	Dimensional outcomes Untreated group	Dimensional outcomes Treated group 1	Dimensional outcomes Treated group 2	Dimensional outcomes Treated group 3
Vance et al., 2004	-	ABX covered by collagen membrane	DFDBA with a putty carrier covered with a CaS barrier	-	Change in horizontal ridge width	-	- 0.5 ± 0.8 mm	- 0.5 ± 0.8 mm	-
					Change in vertical ridge width at the mid-buccal aspect	-	0.7 ± 1.2 mm	- 0.3 ± 0.7 mm	-
					Change in vertical ridge width at the mid-lingual aspect	-	- 0.1 ± 0.8 mm	- 0.5 ± 0.7 mm	-
					Change in vertical ridge width at the mesial aspect	-	- 0.5 ± 0.5 mm	- 0.2 ± 0.6 mm	-
					Change in vertical ridge width at the distal aspect	-	- 0.7 ± 0.8 mm**	- 0.1 ± 0.7 mm	-
*Timepoint of analyses: 4 months*									
Mardas et al., 2010	-	ABX covered by a resorbable bi-layer collagen barrier	SBC covered by a resorbable bi-layer collagen barrier	-	Change of the bucco-lingual/palatal width of the alveolar ridge	-	- 2.1 ± 1.0 mm**	- 1.1 ± 1.0 mm	-
*Timepoint of analyses: 8 months*									
Nam et al., 2011	-	ABX covered by collagen membrane	ABX coated with collagen-binding peptide and covered by collagen membrane	-	Change of the horizontal ridge width	-	- 1.3 ± 1.4 mm	- 1.2 ± 1.5 mm	-
					Change in the height of the buccal crest	-	- 2.3 ± 2.1 mm	- 2.3 ± 3.6 mm	-
					Change in the height of the lingual crest	-	- 1.7 ± 1.9 mm	- 1.1 ± 2.7 mm	-
*Timepoint of analyses: 6 months*									
Gholami et al., 2012	-	ABX spongiosa granules covered by collagen membrane	NCHA covered by collagen membrane	-	Horizontal alveolar ridge width change	-	- 1.07 ± 0.97 mm	- 0.93 ± 0.57 mm	-
*Timepoint of analyses: 6–8 months*									
Cook and Mealey, 2013	-	Collagenated ABX covered by a collagen membrane	Bovine collagen coated with 30% non-sintered HA mineral	-	Change in ridge width	-	- 1.57 ± 1.21 mm	- 1.16 ± 1.44 mm	-
					Change in buccal ridge height	-	- 0.14 ± 2.21 mm	0.03 ± 2.81 mm	-
					Change in lingual ridge height	-	- 0.21 ± 3.04 mm	- 1.18 ± 1.93 mm	-
*Timepoint of analyses: 21 weeks*									
Jung et al., 2013	Spontaneous healing	Collagenated ABX at the bone level and application of a collagen matrix	Collagenated ABX at the bone level and application of an autogenous soft tissue punch graft at the soft tissue level	β-tricalcium-phosphate-particles with polylactid coating without any further treatment at the soft tissue level	Mean change in ridge height at the lingual aspect	- 0.6 ± 0.6 mm	- 0.4 ± 1.4 mm***	- 0.3 ± 1.1 mm*,***	- 1.7 ± 0.6 mm*
					Mean change in ridge height at the buccal aspect	- 0.5 ± 0.9 mm	- 0.0 ± 1.2 mm***	- 1.2 ± 2.9 mm***	- 2.0 ± 2.4 mm
					Mean ridge width change at 1 mm below the most coronal aspect of the crest	- 3.3 ± 2.0 mm	- 1.2 ± 0.8 mm*,***	- 1.4 ± 1.0 mm*,***	- 6.1 ± 2.5 mm*
					Mean ridge width change at 3 mm below the most coronal aspect of the crest	- 1.7 ± 0.8 mm	- 0.6 ± 0.6 mm*,***	- 0.6 ± 0.5 mm*,***	- 3.1 ± 1.6 mm
					Mean ridge width change at 5 mm below the most coronal aspect of the crest	- 0.8 ± 0.5 mm	- 0.1 ± 0.2 mm*,***	- 0.6 ± 0.9 mm***	- 5.7 ± 3.0 mm
*Timepoint of analyses: 6 months*									
Calasans-Maia et al., 2014	-	Bovine ABX type I	Bovine ABX type II	-	Change in horizontal ridge width	-	- 0.39 ± 0.14 mm	- 0.29 ± 0.14 mm	-
*Timepoint of analyses: 6 months*									
Cardaropoli et al., 2014	Spontaneous healing	Collagenated ABX covered by a porcine collagen membrane	-	-	Change in the horizontal width of the alveolar ridge	- 4.04 ± 0.69 mm	- 0.71 ± 0.91 mm*	-	-
					Change in vertical ridge at the mid-buccal site	1.67 ± 0.43 mm	0.56 ± 0.45 mm*	-	-
*Timepoint of analyses: 4 months*									
Pang et al., 2014	Spontaneous healing	ABX covered by absorbable collagen membrane	-	-	Alveolar ridge width change	- 2.72 ± 0.19 mm (3 mo) - 3.56 ± 0.28 mm (6 mo)	- 1.11 ± 0.13 mm* (3 mo) - 1.84 ± 0.35 mm* (6 mo)	-	-
					Alveolar ridge height change	- 2.12 ± 0.15 mm (3 mo) - 3.26 ± 0.29 mm (6 mo)	- 1.05 ± 0.24 mm* (3 mo) - 1.54 ± 0.25 mm* (6 mo)	-	-
*Timepoint of analyses: 3 and 6 months*									
Scheyer et al., 2016	-	Collagenated ABX plus native, bilayer collagen membrane	Demineralized allograft plus reconstituted and cross-linked collagen membrane	-	Horizontal (buccal-lingual) ridge preservation	6.71 ± 2.07 mm**	4.95 ± 2.65 mm	-	-
					Vertical (buccal) ridge preservation	6.24 ± 2.98 mm	5.29 ± 3.73 mm	-	-
					Vertical (lingual) ridge preservation	0.60 ± 2.68 mm	- 0.07 ± 3.15 mm	-	-
*Timepoint of analyses: 6 months*									
Iorio-Siciliano et al., 2017	Spontaneous healing	Collagenated ABX covered by a collagen membrane	-	-	Width change at the buccal-palatal aspects	- 2.8 ± 1.1 mm	- 1.6 ± 1.3 mm*	-	-
					Vertical bone resorption at the buccal aspect with < 1 mm thickness of the buccal bone wall	- 1.7 ± 0.6 mm	- 0.3 ± 0.5 mm*	-	-
					Vertical bone resorption at the linguo-palatal aspect with < 1 mm thickness of the buccal bone wall	- 1.3 ± 0.6 mm	- 0.2 ± 0.4 mm*	-	-
					Horizontal alveolar bone resorption with < 1 mm thickness of the buccal bone wall	- 3.3 ± 0.6 mm	- 2.2 ± 1.3 mm*		
					Vertical bone resorption at the buccal aspect with > 1 mm thickness of the buccal bone wall	- 0.9 ± 1.1 mm	- 0.3 ± 0.5 mm	-	-
					Vertical bone resorption at the linguo-palatal aspect with > 1 mm thickness of the buccal bone wall	- 0.4 ± 0.5 mm	0.0 ± 0.0 mm	-	-
					Horizontal alveolar bone resorption with > 1 mm thickness of the buccal bone wall	- 2.6 ± 1.3 mm	- 0.8 ± 1.0 mm	-	-
*Timepoint of analyses: 6 months*									
Lim et al., 2017	-	Collagenated bovine ABX covered by non-cross-linked collagen membrane	Collagenated porcine ABX covered by cross-linked collagen membrane	-	Horizontal change of alveolar ridge at the 1-mm level	-	- 1.5 ± 1.9 mm (ITT) - 1.5 ± 0.9 mm (PP)	- 1.3 ± 1.6 mm (ITT) - 1.2 ± 0.5 mm (PP)	-
					Horizontal change of alveolar ridge at the 3-mm level	-	- 1.2 ± 0.7 mm (ITT) - 1.2 ± 0.7 mm (PP)	- 1.2 ± 0.7 mm (ITT) - 1.2 ± 0.7 mm (PP)	-
					Horizontal change of alveolar ridge at the 5-mm level	-	- 0.9 ± 0.9 mm (ITT) - 0.9 ± 0.9 mm (PP)	- 0.9 ± 0.6 mm (ITT) - 0.9 ± 0.7 mm (PP)	-
					Vertical change of alveolar ridge at the mesial aspect	-	- 0.7 ± 1.7 mm (ITT) - 0.7 ± 1.7 mm (PP)	- 1.1 ± 1.3 mm (ITT) - 1.3 ± 1.4 mm (PP)	-
					Vertical change of alveolar ridge at the distal aspect	-	- 0.6 ± 1.1 mm (ITT) - 0.6 ± 1.1 mm (PP)	- 0.9 ± 1.6 mm (ITT) - 0.9 ± 1.8 mm (PP)	-
					Vertical change of alveolar ridge at the midfacial aspect	-	- 0.7 ± 1.8 mm (ITT)** - 0.7 ± 1.8 mm (PP)**	- 1.1 ± 2.8 mm (ITT) - 1.5 ± 3.0 mm (PP)	-
					Vertical change of alveolar ridge at the midlingual aspect	-	- 0.2 ± 1.7 mm (ITT) - 0.2 ± 1.7 mm (PP)	- 0.1 ± 2.0 mm (ITT) - 0.1 ± 2.2 mm (PP)	-
*Timepoint of analyses: 4 months*									
Nart et al., 2017	-	ABX covered by a collagen membrane	Collagenated ABX covered by a collagen membrane	-	Change of buccal height	-	- 0.61 ± 0.77 mm	- 0.98 ± 1.28 mm	-
					Change of lingual height	-	- 0.65 ± 0.65 mm	- 0.82 ± 0.61 mm	-
					Change of ridge width at the 1-mm level	-	- 0.91 ± 1.35 mm	- 1.53 ± 1.53 mm	-
					Change of ridge width at the 3-mm level	-	- 0.358 ± 0.31 mm	- 0.788 ± 0.76 mm	-
					Change of ridge width at the 5-mm level	-	- 0.065 ± 0.172 mm**	- 0.16 ± 0.76 mm	-
*Timepoint of analyses: 5 months*									
Pang et al., 2017	-	ABX	Autogenous demineralized dentin matrix	-	Vertical bone gain	-	6.56 ± 3.54 mm	5.38 ± 2.65 mm	-
*Timepoint of analyses: 6 months*									
Serrano Mendez et al., 2017	-	Collagenated ABX covered by collagen membrane	DFDBA covered by collagen membrane	-	Horizontal dimensional changes	-	- 2.6 ± 1.4 mm	- 1.4 ± 1.1 mm	-
					Vertical dimensional changes of the alveolar ridge at mesial aspect	-	- 1.1 ± 1.0 mm	- 0.6 ± 1.3 mm	-
					Vertical dimensional changes of the alveolar ridge at center aspect	-	- 0.4 ± 1.3 mm	0.5 ± 1.4 mm	-
					Vertical dimensional changes of the alveolar ridge at distal aspect	-	- 0.9 ± 1.0 mm	- 0.1 ± 1.4 mm	-
*Timepoint of analyses: 6 months*									
Fischer et al., 2018	Spontaneous healing	ABX	ABX covered by a soft tissue punch from the palate	ABX covered by a resorbable collagen membrane	Change in volumetric buccal ridge contour	- 2.151 ± 1.349 mm	- 0.968 ± 0.344 mm	- 0.874 ± 0.713 mm	- 1.26 ± 0.942 mm
*Timepoint of analyses: 6 months*									
Shim et al., 2018	-	ABX	Hydroxyapatite synthetic bone with rhBMP-2	-	Change in alveolar bone height	-	- 0.20 ± 0.29 mm	1.41 ± 2.28 mm	-
					Change in alveolar bone width	-	- 0.94 ± 1.04 mm**	0.30 ± 1.03 mm	-
*Timepoint of analyses: 3 months*									
Tomasi et al., 2018	-	Collagenated ABX covered by a collagen membrane stabilized by a suture	Blood clot covered by a collagen membrane stabilized by a suture	-	Horizontal change in the ridge at the buccal aspect, measured 2 mm apical of the marginal crest	-	- 1.8 ± 0.9 mm	- 1.5 ± 0.9 mm	-
					Horizontal change in the ridge at the buccal aspect, measured at 4 mm apical of the marginal crest	-	- 1.4 ± 0.9 mm	- 1.2 ± 2.1 mm	-
					Vertical change in the ridge determined at the buccal aspect	-	- 0.2 ± 0.8 mm	- 0.4 ± 0.6 mm	-
					Horizontal change in the ridge at the palatal/lingual aspect, measured at 2 mm apical of the marginal crest	-	- 1.7 ± 0.7 mm	- 1.6 ± 1.0 mm	-
					Horizontal change in the ridge at the palatal/lingual aspect, measured at 4 mm apical of the marginal crest	-	- 1.5 ± 0.5 mm	- 1.2 ± 0.5 mm	-
					Vertical change in the ridge determined at the palatal/lingual aspect	-	- 0.7 ± 0.5 mm	- 0.7 ± 0.7 mm	-
*Timepoint of analyses: 6 months*									
Cha et al., 2019	Extraction alone	Collagenated ABX covered by a collagen membrane	-	-	Change in sinus floor level	- 1.16 mm	- 0.14 mm*	-	-
					Change in bone crest level	- 3.17 mm	0.16 mm*	-	-
					Residual bone height	- 1.98 mm	0.30 mm*	-	-
*Timepoint of analyses: 6 months*									
Lim et al., 2019	Spontaneous healing	Collagenated ABX	Collagenated ABX covered by a native bilayer collagen membrane	-	Change in horizontal ridge width at 1 mm level below the ridge crest	- 4.44 ± 3.71 mm	- 2.49 ± 3.34 mm	- 1.02 ± 0.88 mm*	-
					Change in horizontal ridge width at 3 mm level below the ridge crest	- 2.27 ± 1.15 mm	- 1.17 ± 1.33 mm	- 0.31 ± 1.51 mm*	-
					Change in horizontal ridge width at 5 mm level below the ridge crest	- 0.84 ± 0.75 mm	- 0.59 ± 0.98 mm	0.04 ± 1.29 mm	-
					Change in the vertical height of ridge at buccal crest	- 1.33 ± 1.11 mm	- 1.06 ± 1.57 mm	- 0.58 ± 0.53 mm	-
					Change in the vertical height of ridge at mid crest	-	- 1.15 ± 1.63 mm**	- 0.25 ± 0.95 mm	-
					Change in the vertical height of ridge at lingual crest	- 1.20 ± 0.96 mm	- 0.33 ± 0.38 mm	- 0.12 ± 1.10 mm	-
*Timepoint of analyses: 4 months*									
Llanos et al., 2019	-	ABX covered by a collagen matrix	Collagenated ABX covered by a collagen matrix	-	Change in the horizontal ridge width 1 mm below the buccal alveolar crest	-	- 1.37 ± 0.84 mm	- 1.60 ± 0.82 mm	-
					Change in the horizontal ridge width 3 mm below the buccal alveolar crest	-	- 0.84 ± 0.62 mm	- 0.98 ± 0.67 mm	-
					Change in the horizontal ridge width 5 mm below the buccal alveolar crest	-	- 0.56 ± 0.48 mm	- 0.67 ± 0.47 mm	-
*Timepoint of analyses: 4 months*									
Machtei et al., 2019	Spontaneous healing	ABX	Biphasic calcium sulfate with hydroxyapatite	-	Vertical ridge change	- 1.71 ± 0.4 mm	- 0.25 ± 0.2 mm*	- 0.65 ± 0.5 mm*	-
					Change in horizontal width 3 mm apical to the bone crest	- 2.96 ± 0.3 mm	- 1.56 ± 0.4 mm*,**	- 0.5 ± 0.4 mm*	-
					Change in horizontal width 6 mm apical to the bone crest	- 1.81 ± 0.3 mm	- 0.56 ± 0.4 mm	- 0.81 ± 0.4 mm	-
*Timepoint of analyses: 4 months*									
Santana et al., 2019	-	ABX covered by a PEG barrier membrane	Blood coagulum covered by a PEG barrier membrane	AlloGraft covered by a PEG barrier membrane	Change in ridge width	-	- 2.5 mm	- 2.3 mm	- 1.5 mm
					Change in ridge height at the buccal aspect	-	- 0.23 mm	0.08 mm	- 0.38 mm
					Change in ridge height at the lingual aspect	-	- 0.77 mm	- 0.65 mm	- 0.77 mm
					Change in ridge height at the central aspect	-	12.38 mm	9.93 mm	10.54 mm
*Timepoint of analyses: 6 months*									
Taschieri et al., 2019	-	ABX covered by a palatal graft	70% MgHA + 30% equine collagen	-	Horizontal change of the alveolar ridge	-	- 1.99 ± 0.31 mm	- 2.1 ± 0.90 mm	
									-
					Vertical change of the alveolar ridge at the buccal side	-	- 1.4 ± 0.34 mm	- 1.5 ± 0.30 mm	-
					Vertical change of the alveolar ridge at the lingual site	-	0.41 ± 0.38 mm	0.70 ± 0.30 mm	-
					Crest vertical change	-	1.39 ± 0.31 mm	1.49 ± 0.20 mm	-
*Timepoint of analyses: 6 months*									
Iorio-Siciliano et al., 2020	Spontaneous healing	ABX and collagen membrane	Collagenated ABX and collagen membrane	-	Change in horizontal alveolar ridge width	- 2.3 ± 1.6 mm	- 2.4 ± 1.6 mm	- 2.8 ± 1.4 mm	-
					Vertical changes at buccal aspect	- 2.07 ± 1.94 mm	- 2.92 ± 2.90 mm	- 2.75 ± 1.36 mm	-
*Timepoint of analyses: 6 months*									
Lai et al., 2020	-	Bovine ABX covered by a d-PTFE membrane	Porcine ABX covered by a d-PTFE membrane	-	Increase in lingual ridge height	-	1.56 ± 1.75 mm	1.60 ± 1.74 mm	-
					Change of ridge width	-	- 0.38 ± 1.23 mm	- 1.03 ± 1.3 mm	-
*Timepoint of analyses: 18–20 weeks*									
Lee and Jeong, 2020	Spontaneous healing	Collagenated ABX covered by resorbable collagen membrane	Collagenated ABX + EMD covered by resorbable collagen membrane	-	Horizontal width change at 1 mm apically below the alveolar ridge crest	- 2.36 ± 0.91 mm	- 1.42 ± 0.26 mm*	- 1.44 ± 0.54 mm*	-
					Horizontal width change at 3 mm apically below the alveolar ridge crest	- 2.10 ± 0.53 mm	- 1.34 ± 0.72 mm*	- 1.21 ± 0.52 mm*	-
					Horizontal width change at 5 mm apically below the alveolar ridge crest	- 1.04 ± 0.89 mm	- 0.50 ± 0.61 mm	- 0.86 ± 0.81 mm	-
					Vertical height change in the buccal alveolar ridge crest	- 1.30 ± 0.99 mm	- 0.90 ± 0.65 mm	- 0.61 ± 0.40 mm	-
					Vertical height change in the palatal alveolar ridge crest	- 0.98 ± 0.58 mm	- 0.60 ± 0.37 mm	- 0.69 ± 0.62 mm	-
*Timepoint of analyses: 5 months*									

*Abbreviations*: *ABX* Anorganic bovine xenograft, *ARP* Alveolar ridge preservation, *CaS* Calcium sulphate, *DFDBA* Demineralized freeze-dried cortical bone allograft, *d-PTFE* Dense polytetrafluoroethylene, *EMD* Enamel matrix derivative, *HA* Hydroxyapatite, *ITT* Intention-to-treat, *Mg/HA* Magnesium enriched-hydroxyapatite, *mo* Months, *NCHA* Nanocrystalline hydroxyapatite, *PEG* Polyethylene glycol, *PP* Per protocol, *SBC* Straumann Bone Ceramics®

^*^Significantly different from the untreated group (*p* < 0.05)

^**^Significantly different from the treated group 2 (*p* < 0.05)

^***^Significantly different from the treated group 3 (*p* < 0.05)

**Table 3:** Dimensional changes of the alveolar ridge after ARP procedures with CBXs. Data are presented as Mean ± SD

Reference	Untreated group	Treated group 1 (ARP)	Treated group 2 (ARP)	Description of the endpoint	Dimensional outcomes Untreated group	Dimensional outcomes Treated group 1	Dimensional outcomes Treated group 2
Barone et al., 2008	Extraction alone	CBX covered by a collagen membrane	-	Change in horizontal ridge width	- 4.5 ± 0.8 mm	- 2.5 ± 1.2 mm*	-
				Change in vertical ridge height at mid-buccal aspect	- 3.6 ± 1.5 mm	- 0.7 ± 1.4 mm*	-
				Change in vertical ridge height at mid-lingual aspect	- 3.0 ± 1.6 mm	- 0.4 ± 1.3 mm*	-
				Change in vertical ridge height at mesial aspect	- 0.4 ± 1.2 mm	- 0.2 ± 0.8 mm	-
				Change in vertical ridge height at distal aspect	- 0.5 ± 1.0 mm	- 0.4 ± 0.8 mm	-
*Timepoint of analyses: 7 months*							
Barone et al., 2013	Extraction alone	CBX covered by a collagen membrane	-	Change in bone horizontal dimensions	- 3.6 ± 0.72 mm	- 1.6 ± 0.55 mm	-
				Change in bone vertical dimensions at mesial aspect	- 1.0 ± 0.7 mm	- 0.3 ± 0.76 mm	
				Change in bone vertical dimensions at buccal aspect	- 2.1 ± 0.6 mm	- 1.1 ± 0.96 mm	-
				Change in bone vertical dimensions at distal aspect	- 1.0 ± 0.8 mm	- 0.3 ± 0.85 mm	-
				Change in bone vertical dimensions at lingual aspect	- 2.0 ± 0.73 mm	- 0.9 ± 0.98 mm	-
*Timepoint of analyses: 4 months*							
Festa et al., 2013	Extraction alone	CBX associated with a soft cortical membrane	-	Horizontal ridge width change	- 3.7 ± 1.2 mm	- 1.8 ± 1.3 mm*	-
				Change in vertical ridge height at mid-buccal aspect	- 3.1 ± 1.3 mm	- 0.6 ± 1.4 mm*	-
				Vertical ridge height changes at mid-lingual aspect	- 2.4 ± 1.6 mm	- 0.5 ± 1.3 mm*	-
				Vertical ridge height change at mesial aspect	- 0.4 ± 1.2 mm	- 0.3 ± 0.8 mm	-
				Vertical ridge height change at distal aspect	- 0.5 ± 1.0 mm	- 0.4 ± 0.8 mm	-
*Timepoint of analyses: 6 months*							
Barone et al., 2014	-	CBX covered by a collagen membrane in association with a full-tickness mucoperiosteal flap procedure	CBX covered by a collagen membrane in association with flapless procedure	Change in buccal–lingual width	-	- 3.5 ± 0.9 mm**	- 1.7 ± 0.6 mm
				Change in vertical bone level at buccal aspect	-	- 0.6 ± 0.7 mm**	- 1.1 ± 0.9 mm
				Change of vertical bone level at mesial aspect	-	- 0.4 ± 0.5 mm	- 0.3 ± 0.7 mm
				Change of vertical bone level at distal aspect	-	- 0.5 ± 0.6 mm	- 0.3 ± 0.9 mm
				Change in vertical bone level at lingual–palatal aspect	-	- 0.6 ± 0.7 mm	- 0.9 ± 1.0 mm
*Timepoint of analyses: 3 months*							
Barone et al., 2017	Spontaneous healing	Collagenated CBX covered by a collagen membrane	ABX covered by a collagen membrane	Change in buccal–lingual width	- 3.60 ± 0.72 mm	- 0.93 ± 1.26 mm*	- 1.33 ± 0.71 mm*
				Change in vertical bone level at buccal aspect	- 2.10 ± 0.66 mm	- 0.57 ± 1.54 mm*	- 0.30 ± 1.28 mm*
				Change in vertical bone level at lingual–palatal aspect	- 2.03 ± 0.72 mm	- 1.00 ± 1.17 mm*,**	0.67 ± 2.54 mm*
				Change of vertical bone level at mesial–distal aspect	- 0.15 ± 0.38 mm	- 1.08 ± 1.37 mm*	- 0.90 ± 1.26 mm
*Timepoint of analyses: 3 months*							
Marconcini et al., 2018	Spontaneous healing	CBX covered by a collagen membrane	ABX covered by a collagen membrane	Change in marginal bone height	- 0.69 ± 0.43 mm (1y) - 1.30 ± 0.59 mm (2y) - 1.69 ± 0.43 mm (4y)	- 0.53 ± 0.54 mm (1y)* - 0.80 ± 0.36 mm (2y)* - 0.96 ± 0.51 mm (4y)*	- 0.28 ± 0.37 mm (1y)* - 0.60 ± 0.48 mm (2y)* - 0.75 ± 0.37 mm (4y)*
*Timepoint of analyses: 1, 2 and 4 years*							
Roberto et al., 2021	-	CBX covered by a collagen sheet	Collagen sponge	Change in alveolar ridge width	-	- 2.7 ± 0.9 mm**	- 3.9 ± 1.4 mm
*Timepoint of analyses: 2–3 months*							

*Abbreviations*: *1y* 1 year, *2y* 2 years, *4y* 4 years, *ABX* Anorganic bone xenograft, *CBX* Collagen-preserving bone xenograft

^*^Significantly different from the untreated group (*p* < 0.05)

^**^Significantly different from the treated group 2 (*p* < 0.05)

**Table 4:** Histomorphometric analysis on alveolar sockets grafted with ABXs for ARP. Data are presented as Mean percentages ± SD

Reference	Untreated group		Treated group 1 (ARP)		Treated group 2 (ARP)			Treated group 3 (ARP)				Description of the endpoint	Histomorphometric outcomes Untreated group						Histomorphometric outcomes Treated group 1	Histomorphometric outcomes Treated group 2			Histomorphometric outcomes Treated group 3
Carmagnola et al., 2003	Spontaneous healing		ABX		Collagen membrane			-				Lamellar bone	56.1 ± 18.1%						26.0 ± 23.7%	40.1 ± 15.9%			-
												Woven bone	0.5 ± 1.0%						8.4 ± 8.0%	12.9 ± 15.7%			-
												Bone Marrow	43.0 ± 18.0%						26.2 ± 15.9%	46.0 ± 16.7%			-
												Connective tissue	0%						18.1 ± 17.0%	0%			-
												Residual graft particles	-						21.1 ± 20.0%	-			-
*Timepoint of analyses: 1–15 years for Untreated group; 7 months for Treated group 1; 4 months for Treated group 2*																							
Vance et al., 2004	-		ABX covered by collagen membrane		DFDBA with a putty carrier covered with a CaS barrier			-				Vital bone	-						26 ± 20%**	61 ± 9%			-
												Trabecular spaces	-						54 ± 15%**	32 ± 10%			-
												Residual graft particles	-						16 ± 7%**	3 ± 3%			-
*Timepoint of analyses: 4 months*																							
Mardas et al., 2010	-		ABX covered by a resorbable bi-layer collagen barrier		SBC covered by a resorbable bi-layer collagen barrier			-				New bone formation	-						New bone formation was mainly limited in the apical part of the biopsy, where newly formed bone of either the woven or the mature lamellar type was observed in direct contact with the ABX particles	The newly formed bone was observed mainly at the apical part of the biopsy and was mainly woven, with more lamellar bone occurring only in isolated instances			-
*Timepoint of analyses: 8 months*																							
Heberer et al., 2011	Spontaneous healing		Collagenated ABX		-			-				New bone formation	44.21 ± 24.89%						24.40 ± 10.81%*	-			-
												Graft particles	-						14.75 ± 6.98%	-			-
*Timepoint of analyses: 12 weeks*																							
Nam et al., 2011	-		ABX covered by collagen membrane		ABX coated with collagen-binding peptide and covered by collagen membrane			-				New bone	-						5.3 ± 8.3%**	10.4 ± 4.6%			-
												Connective tissue	-						78.3 ± 19.5%	70.8 ± 8.7%			-
												Graft	-						16.4 ± 12.2%	18.7 ± 7.0%			-
*Timepoint of analyses: 6 months*																							
Gholami et al., 2012	-		ABX spongiosa granules covered by collagen membrane		NCHA covered by collagen membrane			-				Total bone	-						27.35 ± 12.39%	28.63 ± 12.53%			-
												Woven bone	-						18.21% of total bone	13.21% of total bone			-
												Bone marrow	-						20.62 ± 9.91%	13.68 ± 8.07%			-
												Residual graft particles	-						52.03 ± 14.70%	57.69 ± 11.85%			-
*Timepoint of analyses: 6–8 months*																							
Cook and Mealey, 2013	-		Collagenated ABX covered by a collagen membrane		Bovine collagen coated with 30% non-sintered HA mineral			-				Vital bone	-						32.83 ± 14.72%**	47.03 ± 9.09%			-
												Connective tissue/other	-						53.73 ± 6.76%	52.97 – 9.09%			-
												Residual graft	-						13.44 ± 11.57%	ND			-
*Timepoint of analyses: 21 weeks*																							
Calasans-Maia et al., 2014	-		Bovine ABX type I		Bovine ABX type II			-				Vital bone	-						19.3 ± 22.5%	33.6 ± 7.1%			-
												Connective tissue	-						49.9 ± 14.0%	32.3 ± 8.8%			-
												Residual graft	-						22.5 ± 7.9%	10.6 ± 16.2%			-
*Timepoint of analyses: 6 months*																							
Milani et al., 2016	Spontaneous healing		ABX covered by a resorbable membrane		-			-				Lamellar bone	30.6 ± 13.4%						14.6 ± 12.2%	-			-
												Osteoid	29.3 ± 11.2%						25.8 ± 22.3%	-			-
												Bone marrow	40.1 ± 28.3%						26.2 ± 14.3%	-			-
												Residual graft material	-						33.4 ± 27.2%	-			-
*Timepoint of analyses: 5 months*																							
Scheyer et al., 2016	-		Collagenated ABX plus native, bilayer collagen membrane		Demineralized allograft plus reconstituted and cross-linked collagen membrane			-				New bone	-						29.81 ± 9.03%	33.36 ± 11.09%			-
												Connective tissue/ bone marrow	-						50.77 ± 8.26%	53.66 ± 7.62%			-
												Graft remnants	-						19.40 ± 10.99%**	12.78 ± 6.60%			-
*Timepoint of analyses: 6 months*																							
Nart et al., 2017	-		ABX covered by a collagen membrane		Collagenated ABX covered by a collagen membrane			-				Newly formed bone	-						33.44 ± 17.82%	37.68 ± 13.38%			-
												Connective tissue	-						53.88 ± 17.43%	50.31 ± 19.20%			-
												Residual graft particles	-						13.14 ± 8.32%	16.00 ± 11.60%			-
*Timepoint of analyses: 5 months*																							
Pang et al., 2017	-		ABX		Autogenous demineralized dentin matrix			-				Newly formed bone	-						35.00 ± 19.33%	31.24 ± 13.87%			-
												Soft tissues	-						47.93 ± 24.46%	59.81 ± 15.50%			-
												Residual graft material	-						17.08 ± 16.57%	8.95 ± 6.15%			-
*Timepoint of analyses: 6 months*																							
Serrano Mendez et al., 2017	-	Collagenated ABX covered by collagen membrane		DFDBA covered by collagen membrane					Newly formed bone -							35.3 ± 16.8%				25.5 ± 10.1%			-
									Marrow spaces -							19.8 ± 10.8%				24.2 ± 16.5%			-
									Total bone volume -							55.0 ± 25.1%				49.7 ± 19.5%			-
									Soft tissue -							22.8 ± 13.7%				16.5 ± 12.1%			-
*Timepoint of analyses: 6 months*																							
Shim et al., 2018	-	ABX		Hydroxyapatite synthetic bone with rhBMP-2			-		New bone -							6.13 ± 4.32%**				25.37 ± 17.23%			-
									Residual graft -							16.79 ± 1.46%				12.03 ± 8.03%			-
*Timepoint of analyses: 3 months*																							
Lim et al., 2019	Spontaneous healing	Collagenated ABX		Collagenated ABX covered by a native bilayer collagen membrane		-				Newly formed bone					25.16 ± 18.45%		11.32 ± 7.39%				16.92 ± 14.86%		-
										Soft tissues (epithelium, connective tissue)					2.79 ± 1.19%		1.97 ± 1.20%				2.09 ± 0.44%		-
										Graft particles					-		16.96 ± 8.93%				11.23 ± 7.64%		-
*Timepoint of analyses: 4 months*																							
Machtei et al., 2019	Spontaneous healing	ABX		Biphasic calcium sulfate with hydroxyapatite		-				Newly formed bone					81.72 ± 4.3%			22.50 ± 24.72%*, **				44.15 ± 18.8%*	
										Residual graft particles					-			40.18 ± 17.2%**				16.51 ± 16.2%	
*Timepoint of analyses: 4 months*																							
Santana et al., 2019	-	ABX covered by a PEG barrier membrane		Blood coagulum covered by a PEG barrier membrane		AlloGraft covered by a PEG barrier membrane					New bone				-			28.18%**				47.81%	33.34%
											Connective tissue				-			62.93%				52.19%	58.43%
											Residual graft particles				-			8.89%				-	8.23%
*Timepoint of analyses: 6 months*																							
Taschieri et al., 2019	-	ABX covered by a palatal graft		70% MgHA + 30% equine collagen		-					Newly formed vital bone				-			22.77 ± 6.95%				23.07 ± 10.3%)	-
											Residual graft particles							15.77 ± 1.95%**				5.01 ± 1.04%	-
*Timepoint of analyses: 6 months*																							
Lai et al., 2020	-	Bovine ABX covered by a d-PTFE membrane		Porcine ABX covered by a d-PTFE membrane		-						Vital bone			-			36.21 ± 26.51%				31.27 ± 16.23%	-
												Connective tissue/other			-			43.32 ± 15.78%				49.21 ± 10.79%	-
												Residual graft material			-			20.47 ± 15.29%				19.52 ± 9.19%	-
*Timepoint of analyses: 18–20 weeks*																							

*Abbreviations*: *ABX* Anorganic bone xenograft, *ARP* Alveolar ridge preservation, *CaS* Calcium sulphate, *DFDBA* Demineralized freeze-dried cortical bone allograft, *HA* Hydroxyapatite, *Mg/HA* Magnesium enriched-hydroxyapatite NCHA, nanocrystalline hydroxyapatite, *ND* Non-detected, *PEG* Polyethylene glycol, *rhBMP-2* Recombinant human bone morphogenetic protein-2, *SBC* Straumann Bone Ceramics®

^*^Significantly different from the untreated group (*p* < 0.05)

^**^Significantly different from the treated group 2 (*p* < 0.05)

**Table 5:** Histomorphometric analysis on alveolar sockets grafted with CBXs for ARP. Data are presented as Mean percentages ± SD

Reference	Untreated group	Treated group 1 (ARP)	Treated group 2 (ARP)	Description of the endpoint	Histomorphometric outcomes Untreated group	Histomorphometric outcomes Treated group 1	Histomorphometric outcomes Treated group 2
Barone et al., 2008	Extraction alone	CBX covered by a collagen membrane	-	Total bone volume	25.7 ± 9.5%	35.5 ± 10.4%*	-
				Connective tissue	59.1 ± 10.4%	36.6 ± 12.6%*	-
				Residual graft material	-	29.2 ± 10.1%	-
*Timepoint of analyses: 7 months*							
Barone et al., 2015	-	CBX covered by a collagen membrane and added with a full thickness mucoperiosteal flap and primary soft tissue closure	CBX covered by a collagen membrane with a flapless procedure and a secondary soft tissue closure	Newly formed bone	-	22.5 ± 3.9%	22.5 ± 4.3%
				Marrow spaces	-	59.3 ± 7.5%	59.4 ± 6.8%
				Residual graft material	-	18.2 ± 6.1%	18.2 ± 5.2%
*Timepoint of analyses: 3 months*							
Barone et al., 2017	Spontaneous healing	Collagenated CBX covered by a collagen membrane	ABX covered by a collagen membrane	Newly formed bone	44.0 ± 14.7%	41.4 ± 20.6%	36.8 ± 19.1%
				Non-mineralized tissues	56.0 ± 14.7%	41.4 ± 15.9%*	47.8 ± 19.2%
				Residual graft particles	-	14.9% ± 7.3%	15.5 ± 8.4%
*Timepoint of analyses: 3 months*							
Di Stefano et al., 2019a	-	CBX covered by a collagen membrane	ABX covered by a collagen membrane	Newly formed bone	-	45.12 ± 10.54%**	33.61 ± 9.71%
				Residual biomaterial	-	10.91 ± 4.27%**	18.47 ± 5.62%
*Timepoint of analyses: 4–8 months*							

*Abbreviations*: *ABX* Anorganic bone xenograft, *CBX* Collagen-preserving bone xenograft

^*^Significantly different from the untreated group (*p* < 0.05)

^**^Significantly different from the treated group 2 (*p* < 0.05)

### Study characteristics

An overview of the main characteristics of eligible papers is provided by Table 1. Most studies (*n* = 30) resulted to be randomized controlled trials (RCT), with either prospective (*n* = 7) and retrospective (*n* = 2) clinical trials being selected during the literature search. Almost all the studies considered tooth extraction, ARP procedures and delayed implant placement as surgical interventions. Besides the primary outcome variables, site eligibility, histological healing characteristics, and complication were the most frequently reported secondary outcomes.

### Risk of bias assessment

**Fig. 2:**
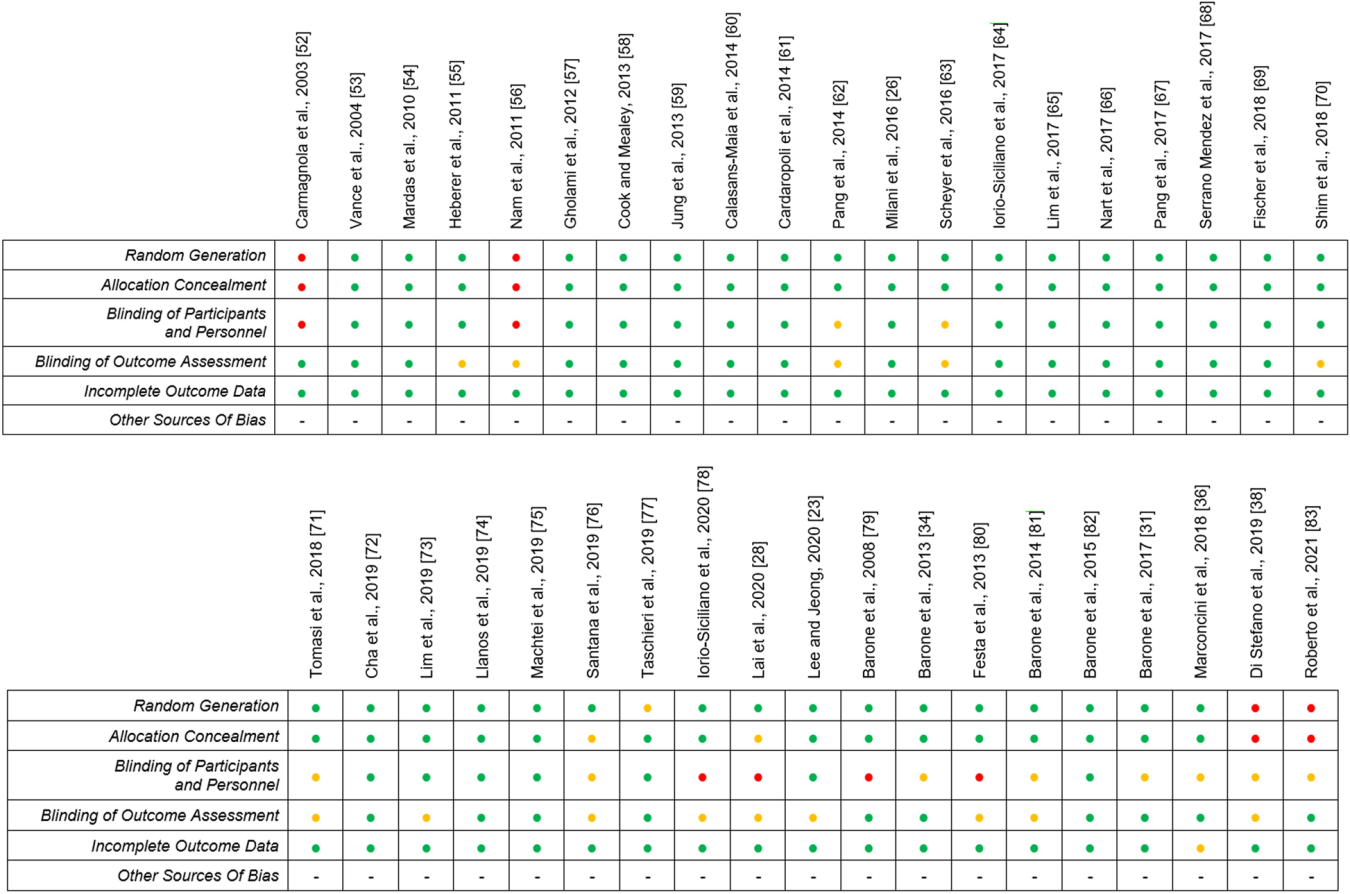
Risk of bias assessment for the qualitative evaluation of the studies included in the systematic review. For each study, low (green), uncertain (yellow), or high risk (red) of bias was assessed according to the presence of established quality criteria

Considering the quality criteria listed in Paragraph "Risk of bias assessment" of the Materials and Methods section, each study was classified into one of the following groups: “low risk of bias”, when all quality criteria were considered to be “present”, “moderate risk of bias”, when one or more key domains were “unclear”, and “high risk of bias”, when one or more quality criteria were “absent”. Results of risk of bias assessment are described in Fig. 2. Overall, the analysis revealed good quality of the selected studies, with major concerns regarded Blinding of Participants and Personnel and blinding of outcome assessment, which were unclearly reported or missing in some trials.

### ABXs versus CBXs: dimensional changes

#### Bone xenografts vs. spontaneous healing

Most reviewed clinical trials that compared spontaneously healed alveoli and the filling of post-extraction sockets with anorganic bone-based grafts reported significantly less horizontal and vertical bone resorption with the ARP procedure (Table 2) [23, 59, 61, 62, 64, 72, 73, 75, 84]. Conversely, other trials identified no significant differences among grafted and non-grafted sites [58, 69], while stating clinical relevance of ABX-based ARP in esthetically demanding cases [69] or suggesting no significant benefits of the treatment in post-extraction sites with good alveolar bone wall integrity and adequate buccal bone wall thickness [58].

Regarding collagen-preserving materials, a positive trend was recognized about the preservation of bone dimensions of post-extraction sites by clinical trials which compared spontaneously healed sockets with the grafting of CBXs associated with a collagen membrane [34, 79] or soft cortical lamina [80] (Table 3). Specifically, the ridge-preservation treatment showed to significantly reduce the resorption of horizontal ridge width and vertical ridge height at mid-buccal and mid-lingual aspects in comparison with extraction alone [79, 80]. Moreover, even when no significant differences between CBXs-treated and untreated groups were detected, less resorption of hard tissue ridge (both horizontal and vertical dimensions) was measured in grafted sites [34].

#### Modified bone xenografts

Association of ABXs with additional conditions/treatments has been investigated in the effort to enhance the preservation of ridge dimensions in post-extraction sockets. For instance, a composite xenograft consisting of 90% anorganic bovine bone embedded in a 10% biodegradable collagen matrix of porcine origin has been widely investigated in comparison or in substitution of ABXs  alone to minimize bone dimensional changes after tooth extraction [23, 58, 59, 61, 61, 63–66, 68, 71–74, 78]. In this composite, collagen facilitates graft handling and ameliorates graft adaptation and stabilization to the defect, with pre-clinical data establishing that collagenated anorganic bone serves as a scaffold for bone formation rather than promoting tissue regeneration [85]. However, existing clinical evidence revealed non-inferiority of ABXs compared to collagenated ABXs, except for significantly less reduction in ridge width at the 5-mm level reported by some trials [58, 63, 78, 86]. As demonstrated for porcine collagen addition, the coating of ABXs with synthetic oligopeptide from the collagen-binding domain of osteopontin also showed not to ameliorate ARP outcomes [56] (Table 2).

On the other hand, trials investigating CBXs for ARP procedures never considered to implement them with collagen-derived additives, probably due to the fact that these grafting materials contain a more preserved collagenic component.

#### Bone xenografts associated with barrier membranes

As an aid for alveolar ridge preservation, the use of barrier membranes in combination with bone grafts during ARP procedures was demonstrated to prevent epithelial downgrowth into the alveolar socket, whereas the graft material avoids membrane collapse and promotes bone formation through osteoconduction and/or osteoinduction processes [87]. Resorbable collagen matrices are the membranes of choice to cover ABXs-grafted sockets, with conflicting outcomes reported by clinical trials which demonstrated both (a) the effective reduction of horizontal ridge changes, with significant preservation of vertical height at mid crest [73] and (b) failure to limit the loss of horizontal/vertical ridge dimensions in comparison with the application of collagen membrane without ABXs [74]. Moreover, the addition of an enamel matrix derivative (EMD) to collagenated ABXs covered with a collagen membrane did not showed significant improvement of ridge preservation compared to the EMD-lacking group, although horizontal width changes were significantly greater in the non-grafted sockets compared with both types of grafted sites [23].

Concerning CBXs, all selected trials evaluating ridge dimension outcomes described the graft covering with collagen membranes [31, 34, 36, 79, 81, 83, 88] or with a cortical bone-derived lamina [80], not even considering the CBXs alone (Table 3). Interestingly, Barone and colleagues [81] investigated the clinical effects on coupling CBXs grafts covered by a collagen matrix with a full flap procedure to cover the membrane, or a flapless procedure leaving the membrane exposed. More successful preservation of horizontal ridge dimension was assured by the flapless procedure, with additional advantages given by the positive increase in keratinized gingiva.

Besides collagen membranes, both natural and synthetic materials were tested to cover the ABX. For instance, autogenous soft tissue punches from the palate were used to cover ABXs or collagenated ABXs particles in post-extractive sockets, assuring for significantly less resorption of vertical and horizontal ridges with respect to spontaneous healing, but not to the use of collagen matrix (Table 2) [59, 69]. Interestingly, the application of a synthetic polyethylene glycol (PEG) barrier both alone or in association to ABX was reported to be effective in preventing vertical bone loss at the buccal/lingual aspects and even promoting vertical bone gain at the central aspect (Table 2) [76].

#### Bone xenografts vs. allogenic or autologous grafts

Considering clinical outcomes achieved by ABXs versus allograft materials, conflicting results are currently found in the literature. On the one hand, collagenated ABXs were reported to preserve the horizontal alveolar ridge dimension significantly better than allogenic materials, providing more bony width at the grafted site [65]. Conversely, no statistically significant differences in horizontal and vertical bone changes were found by a more recent RCT comparing collagenated ABXs with allogenic material [71]. Additionally, some clinical evidence even attested the superiority of bone allografts over ABXs to prevent horizontal [76] or vertical [53] bone loss after tooth extraction.

Regarding the comparison with autologous grafts, autogenous demineralized dentin matrix was demonstrated to be as effective as ABXs for augmenting vertical bone dimensions after tooth extraction [67].

Considering CBXs, no clinical comparisons with allogenic/autologous graft materials were investigated so far, this representing a significant gap of knowledge about the efficacy of these bone xenografts for ARP procedures.

#### Bone xenografts vs. synthetic grafts

Clinical trials investigating the effects of ABXs versus synthetic materials on ridge preservation described equivalent clinical efficacy in controlling horizontal/vertical resorption when comparing the bone xenograft and nanocrystalline hydroxyapatite (HA) [57] or HA-collagen composites [66, 77]. On the contrary, better outcomes were exhibited by the synthetic counterpart when anorganic bone was compared with biphasic calcium sulphate/hydroxyapatite (BCS/HA) [75], HA treated with recombinant human bone morphogenetic protein-2 (rhBMP-2/HA) [70] and a biphasic ceramic bone substitute made of HA and β-tricalcium phosphate (β-TCP) (i.e., Straumann Bone Ceramics-SBC) [54]. Unlike aforementioned studies, clinical evidence was reported about the significant superiority of collagenated ABXs over β-TCP particles with polylactide coating in limiting ridge height and width changes after tooth extraction (Table 2) [59].

As previously described for the comparison with allogenic/autologous grafts, no clinical trials evaluated the dimensional outcomes of CBXs *vs*. synthetic material grafting during ARP procedures.

#### Comparison among different heterologous graft materials

Some clinical trials compared anorganic bone from different species, demonstrating that alternative sources of ABXs can be used with comparable outcomes. Overall, anorganic bovine and porcine bone grafts were found to be equally effective in reducing horizontal ridge changes in post-extraction sockets, with anorganic porcine material showing significantly lower efficacy in vertical ridge preservation [68] and more frequent failure of implant stability [28]. Following this trend, two deproteinized bovine bone minerals were demonstrated to be comparable in preserving horizontal ridge width, affording a more favorable implant position [60].

Recently, CBXs and ABXs in combination with a collagen membrane were compared for alveolar ridge preservation, along with natural healing of the post-extraction sockets [31, 36] (Table 3). A significantly lower reduction of buccal-lingual width and vertical bone dimensions was registered at the grafted sockets compared to non-grafted sites, with ABXs being significantly more effective than CBXs in preserving vertical bone level at the lingual–palatal aspect [31]. On the contrary, the trial by Marconcini and collaborators [36] detected no significant differences between the two grafting materials regarding peri-implant crestal bone loss, which was significantly greater in the non-grafted sockets at each follow-up period (1, 2, and 4 years). Ridge preservation was also significantly more effective than spontaneous healing in peri-implant soft tissue recovery, with ABXs showing better aesthetic outcomes than CBXs [36].

Finally, CBXs were also shown to be significantly more effective than collagen sponges to preserve alveolar ridge width measured soon after tooth extraction and 2–3 months post-grafting with the two biomaterials. Specifically, changes in alveolar width were not significant in premolar sites, but significant differences were observed between the two graft procedures at molar sites [83].

### ABXs versus CBXs: histomorphometric evaluation

Overall, histological investigations of extraction sockets grafted with ABXs or CBXs showed no signs of adverse reaction or severe inflammatory response towards the heterologous bone substitute suggesting that anorganic bone [55] and CBXs of both porcine [31, 79, 82], and equine [38] origin are safe and biocompatible ARP biomaterials.

#### Bone xenografts vs. spontaneous healing

Compared with alveolar sockets left to heal spontaneously, ABXs [26, 73] and CBXs [31, 79] exhibited comparable [26, 31, 73] or even improved [79] histomorphometric outcomes at the grafted site regarding new bone formation or soft tissue amount (Tables 4 and 5). Conversely, Heberer and collaborators [55] provided evidence of a significantly lower rate of new bone formation in the anorganic bone-filled sockets in comparison with non-grafted sites. Bone apposition was observed in the proximity of ABXs particles, but resorptive processes were absent. Additionally, a significantly higher amount of NFB was detected in the apical rather than the coronal region of the extraction site, regardless of the grafting procedure, suggesting that bone formation could be initiated from the apical/lateral region of the alveolar socket and was not enhanced from the coronal direction [55, 89]. These results are in line with evidence previously reported by Carmagnola and colleagues [52], who demonstrated that anorganic bone grafting led to less new bone formation and more residual connective tissue compared with cases where graft materials were not used, although no statistical analysis was performed to prove significant differences.

#### Modified bone xenografts

Concordant with clinical data regarding bone dimensional changes in post-extraction sockets, histomorphometric evaluations demonstrated that collagenated ABXs did not enhance newly formed bone (NFB) in comparison with ABXs [63] (Table 4). In general, ABXs particles were found to be surrounded more by new vital bone rather than connective tissue, but no signs of particle resorption were observed. These results support animal studies reporting that ABXs elimination might be very slow or even remain unaltered in the osseous tissue [85]. Unlike addition of the collagen carrier, coating the ABXs with collagen-binding peptide significantly affected the percentage of NFB in the extraction socket compared to uncoated ABXs [56] (Table 4). Histological and histomorphometric investigations highlighted new bone formation both at the periphery and in the central/coronal regions with direct bone apposition over the graft surface, indicating high osteoconductive and osteoinductive effects, with improved biocompatibility of the peptide-modified ABXs proven by the significantly higher bone-to-graft contact in comparison with unmodified ABXs [56].

#### Bone xenografts associated with barrier membranes

In ARP procedures, biological/synthetic resorbable membranes are used to accelerate bone formation by preventing the ingrowth of connective or epithelial tissue [90]. Histomorphometric analysis of post-extraction sockets grafted with collagenated ABXs with or without the addition of collagen membrane did not show significantly increased formation of new bone or better biomaterial resorption when the graft particles were covered with the barrier matrix [73] (Table 4). However, in the presence of the collagen membrane, the mean percentages of NFB and residual graft material were higher and lower, respectively [68]. On the other hand, improved histomorphometric outcomes were observed following the application of a PEG membrane to cover ungrafted sockets, with the formation of a significantly higher amount of new bone in comparison with anorganic bone grafts associated with the same device [76] (Table 4).

Similar to clinical evidence collected about ABXs plus collagen matrix, a clinical trial evaluating CBXs covered with collagen membrane and associated to flapless *versus* flap elevation techniques highlighted no significant histological or histomorphometrical differences between the two procedures [82] (Table 5).

#### Bone xenografts vs. allogenic or autologous grafts

Most clinical trials comparing ABXs (± heterologous collagen) and bone allografts highlighted that both materials performed well histologically and resulted in comparable amounts of new bone formation in the grafted sockets [65, 71, 76]. Significantly higher amounts of collagenated ABXs rather than allograft remnants were observed in the grafted sites, confirming a previous hypothesis on the poor resorption rate of the xenograft material. Little or no signs of osteoclastic resorption and graft remodeling were observed, whereas bone allografts histologically exhibited a more active state of turnover and replacement within the grafted socket [65]. Unlike the above cited studies, only one trial reported clear superiority of bone allograft mixed with an experimental putty carrier compared to ABXs in producing significantly more vital bone filling the extraction socket [53] (Table 4).

Finally, statistically significant differences in histomorphometric outcomes were not observed when ABXs were compared to autogenous demineralized dentin matrix for ridge preservation. The graft biomaterials displayed adequate tissue integrity, with both substitutes surrounded by and in direct contact with NFB to confirm their osteoconductive properties [62].

#### Bone xenografts vs. synthetic grafts

Similar to clinical measurements of ridge dimensions, histomorphometric studies showed comparable [54, 57] or inferior [70] performance of ABXs versus synthetic material grafting in the post-extraction socket. In particular, equivalent histological characteristics of biopsies ABXs from - and SBC-treated sockets were found, with NFB mainly localized in the apical region and in direct contact with the graft particles [54]. Similarly, no statistical differences were reported by histomorphometric analyses comparing ABXs and nanocrystalline hydroxyapatite (NCHA) socket grafting [57]. On the other hand, rhBMP-2/HA was found to achieve significantly greater new bone formation than ABXs in treated sockets, whereas comparable outcomes for the two biomaterials were registered for soft tissue and residual graft particles (Table 4). As reported by other histomorphometric studies [54, 55], a stronger tendency to produce new bone in the apical region compared with the coronal portion was evidenced in both treatment conditions [77]. Finally, when collagenated ABXs were compared with HA-collagen composites, a significantly lower percentage of NFB [66] and significantly higher amounts of residual biomaterial particles [77] were histomorphometrically detected within the treated alveolar sockets.

#### Comparison among different heterologous graft materials

Histologically, similar efficacies of anorganic bone from different species were demonstrated. No statistically significant differences were detected among extraction sites treated with bovine and porcine anorganic bone [28] or different deproteinized bovine bone xenografts [60] with regard to the mean percentage of vital bone formation, residual graft material, and connective tissue (Table 4). Both bovine and porcine ABXs showed high porosity that allowed for new bone formation and ingrowth [28].

Three clinical trials reported comparisons between CBXs and ABXs for ARP with conflicting results. On the one hand, no significant differences were detected in terms of NFB, connective tissue prevalence, and residual graft particles in the alveolar socket. Nevertheless, a higher percentage of NFB and lower amount of residual bone substitute were found in the CBXs-treated group, likely indicating different resorption rates for the two biomaterials and possibly a more promising healing pattern for CBXs compared to ABXs [31]. More intriguing histological evidence was recently reported by Di Stefano and colleagues [38]. Besides demonstrating the presence of native type I bone collagen in CBXs, but not in ABXs, this study detected a significantly greater quantity of NFB and fewer residual biomaterial particles after socket grafting with collagen-preserving material rather than anorganic heterologous bone. These findings are the first clinical demonstration that the manufacturing process can greatly affect xenograft behavior, underscoring the importance of preserving bone collagen in its native form to enhance the biomaterial’s regenerative effect (Table 5).

### ABXs versus CBXs: secondary outcome variables

High heterogeneity was found regarding secondary outcome variables reported by the selected clinical trials. A frequently evaluated variable was site elegibility for implant placement after ARP and eventual need for bone augmentation regardless the grafting procedure. Concerning ABXs, several trials reported that both grafted and ungrafted sites healed uneventfully, showing adequate alveolar ridge preservation to receive an implant without any additional grafting or bone augmentation procedure [26, 52, 53, 56, 60, 62, 64, 67, 71, 78]. Conversely, other authors highlighted the need to perform additional augmentation along with dental implant placement due to insufficient ridge volume [28, 63, 65, 69, 74] or to the presence of fenestration or small dehiscence at the grafted site [57, 58, 69]. Remarkably, Cha and collaborators [72] provided evidence supporting more frequent bone augmentation for ungrafted rather than grafted sockets. Similar trends were observed for CBXs, with some trials describing implant placement without the need for bone augmentation in both untreated and treated sockets [79, 82] and other studies reporting better volume conditions for implant loading in grafted sites [34, 36].

Postoperative histological analyses of the healed sockets mostly demonstrated newly formed keratinized mucosa and no signs of inflammation for both ABXs- [26, 28, 54, 55, 70, 77] and CBXs- [38, 79, 82] grafted sites, confirming the biocompatibility of both materials. Also, supporting graft bio-safety, no post-operative complications (i.e., rejection or wound infections around the grafting region) were generally recorded at any surgical site by both ABXs [28, 55, 59–62, 64, 66, 67, 71, 74, 77, 78] and CBXs [36, 38, 79–81] trials.

Among dimensional outcomes, buccal plate thickness was poorly considered by selected clinical studies, although it was proven to affect the amount of horizontal and vertical crest resorption in human sockets [61]. Overall, ABXs trials detected non-significant changes in buccal plate thickness among naturally healed sites and grafted sockets [28, 61, 74, 75], finding a negative correlation between the initial thickness of the buccal bone and ridge width reduction in non-grafted but not in treated alveoli [61, 66]. Different results were reported for CBXs, which was found to lead to buccal cortical plate loss in the long term (10-year follow-up) [83].

Only one trial performed bone volume measures on the post-extraction sockets, demonstrating significantly lower bone resorption in ABXs-treated versus naturally healed sites [62].

Finally, very few studies reported on patient-related outcomes following socket preservation. The severity of pain, discomfort and swelling was assessed in ABXs trials by using the visual analog scale (VAS) score [75, 77] or self-report questionnaires [23, 77], revealing low to moderate pain level following surgery [75] and no significant score differences between grafted and ungrafted patients [23].

## Discussion

The effects of ridge preservation with the use of different biomaterials have been thoroughly investigated, and filling of post-extraction sites with bone xenografts was clinically demonstrated to significantly reduce ridge changes in comparison with spontaneously healed sockets [91]. Ridge preservation treatment also reduced the need for further bone augmentation at the time of implant placement, ameliorating the aesthetic outcome of implant rehabilitation [34, 81]. Xenogenic material currently used for ridge preservation is predominantly anorganic bovine/porcine bone made from the inorganic portion of animal bone tissue. The manufacturing process to produce ABXs is based on high-temperature treatment (> 300 °C), which removes cells and xenogenic antigens to avoid potential immunologic reactions. This method also eliminates all organic components and proteins, while HA with enhanced crystallinity is maintained as the main graft constituent [60, 92, 93]. Deproteinized xenografts were demonstrated to have good physico-chemical and osteoconductive properties in ridge preservation strategies. Nevertheless, suboptimal biointegration and bioabsorption characteristics of heat-treated materials suggest that the processing protocol for xenograft bone substitutes may greatly affect the biomaterial behavior in situ regarding the regenerative potential and quality of NFB [93]. To overcome these limitations, bone xenografts fabricated with less aggressive treatment to remove xenogenic antigens were proposed to preserve the collagen component of the animal bone, ultimately improve the bioactive properties of the final product [38, 94]. The preservation of type I collagen in bone substitutes can improve socket healing in ARP procedures by a series of processes, including (1) enhanced stimulation by endogenous growth factors; (2) longer duration of regenerative stimuli; (3) physiological modulation of bone metabolism and remodeling; and (4) increased osteoblast adhesion, proliferation, and differentiation [95–98]. Indeed, this might have contributed to the successful clinical outcomes with CBXs use reported for different oral surgery procedures including sinus lift bone grafting [42, 99–102], ridge augmentation [103–105], and peri-implant-guided bone regeneration [106–108]. However, direct clinical comparisons between anorganic and CBXs for socket preservation were only reported in three clinical trials [31, 38, 82], so the superiority of one biomaterial over another has not been established yet. In this work, clinical research testing ABXs or CBXs for ridge preservation was systematically reviewed to perform a preliminary comparison in terms of the biomaterials’ dimensional and histomorphometric outcomes. Table 6 summarizes the collected results, presenting minimum and maximum average values and standard deviations recorded for horizontal/vertical ridge resorption, as well as the percentage of NFB, connective tissue, and residual graft particles at the grafted sites.

**Table 6:** Dimensional and histomorphometric clinical outcomes obtained by grafting post-extraction sockets with ABXs *versus* CBXs for ARP

**Type of xenograft for ARP**		**Dimensional change (Min–Max)**	**SD** **(Min–Max)**		**Histomorphometry** **(Min–Max)**	**SD** **(Min–Max)**
**Anorganic** **Bone xenograft**	Horizontal Ridge Resorption	0.065 – 2.8 mm	0.13 – 3.34 mm	Newly Formed Bone	5.3 – 37.68%	4.32 – 26.51%
	Vertical Ridge Resorption	0.1 – 2.92 mm	0.2 – 3.6 mm	Connective Tissue	1.97 – 78.3%	0.44 – 24.46%
				Residual graft material	8.89 – 52.03%	1.46 – 27.2%
**Collagen-preserving** **Bone xenograft**	Horizontal Ridge Resorption	0.93 – 3.5 mm	0.55 – 1.3 mm	Newly Formed Bone	22.5 – 45.12%	3.9 – 20.6%
	Vertical Ridge Resorption	0.2 – 1.1 mm	0.5 – 1.54 mm	Connective Tissue	36.6 – 41.4%	12.6 – 15.9%
				Residual graft material	10.91 – 29.2%	4.27 – 10.1%

Clinical outcomes for alveolar ridge dimensional changes showed successful socket preservation when using both ABXs and CBXs in comparison with spontaneous healing, with ABXs yielding better results than untreated control and largely similar to bone allografts and synthetic materials. Horizontal ridge resorption was calculated to range from 0.065 to 2.8 mm for ABXs and from 0.93 to 3.5 mm for CBXs, with standard deviations ranging from 0.14 to 3.34 mm and from 0.55 to 1.3 mm, respectively. Thus, lower minimum and maximum values of horizontal bone loss were observed for ABXs, but the standard deviations showed a broader value range compared with CBXs (Table 6).

Vertical ridge reduction was found to be between 0.1 and 2.92 mm for ABXs and between 0.2 and 1.1 mm for CBXs, with standard deviations ranging from 0.2 to 3.6 mm and from 0.5 to 1.54 mm, respectively. In this case, ABXs showed a lower minimum change but higher maximum alteration of vertical ridge dimensions with respect to CBXs, but the value range for standard deviation was still broader for the heat-treated bone substitute (Table 6).

Histomorphometric evaluations after ARP of the post-extraction sockets produced less obvious results for the superiority of both anorganic bone substitutes and CBXs over spontaneous healing or other treatments, since significant differences in terms of new bone formation were less frequently reported by clinicians. However, high biocompatibility and capacity to promote bone regeneration were observed for both xenografts. Remarkably, Di Stefano and co-workers [38] provided the first evidence of significantly better histological performance for CBXs rather than ABXs, supporting the hypothesis that maintaining type I collagen in its native conformation may improve the biological effects of the graft and promote faster remodeling of the heterologous material [109].

In summary, despite the much larger number of clinical trials for ABXs rather than CBXs, the two types of xenografts seem to provide overlapping dimensional/histological outcomes with large measurement dispersion, underscoring the need of comparative clinical studies that may demonstrate the superiority of one material over the other at a statistically significant level.

Regarding histomorphometrical measurements, NFB was between 5.3 and 37.68% for ABXs and between 22.5 and 45.12% for CBXs, with standard deviations ranging from 4.32 to 26.51% and from 3.9 to 20.6%, respectively. Based on that, higher amount of NFB and lower variability were registered for CBXs versus ABXs. This trend was also confirmed for data concerning residual graft particles, which overall exhibited better results for CBXs (lower range values, 10.92–29.2%) compared to ABXs (higher range values, 8.89–52.03%), with less variability for the collagen-preserving biomaterials (10.91–29.2% for CBXs and 8.89–52.03% for ABXs) (Table 6). As shown in Table 6, the amount of NFB was on average higher for CBXs rather than ABXs, with the minimum value being much greater (> 17.2%) for CBXs with respect to ABXs. On the other hand, the average amount of residual graft particles was lower for CBXs, which had a clearly inferior maximum value and standard deviation range with respect to ABXs. Regarding connective tissue evaluation, lower measurement dispersion was observed for CBXs in comparison with ABXs. Although these trends need to be verified in controlled clinical studies, they are in line with evidence collected by recent trials that compared ABXs and CBXs and demonstrated better histomorphometric outcomes for CBXs in both ARP [38] and sinus augmentation [42] procedures.

Concerning dimensional outcomes, some possible trends might be hypothesized based on collected data regarding horizontal ridge resorption, which seems to be more limited by anorganic bone grafting, albeit with a larger measurement dispersion (maximum standard deviation for ABXs is about three times higher than for CBXs). Conversely, vertical ridge preservation seems to be well achieved by CBXs, with maximum resorption measures more than halved compared to ABXs (Table 6).

One topic meriting discussion is data variability, which appears high for all the endpoints of interest, both among different studies and within each study included in this review. Variability among studies may be explained by the different surgical techniques and various methods to measure the same endpoints. Endpoints describing dimensions varied: vertical or horizontal width, buccal versus lingual plates, measurements performed at the crestal level or at different vertical levels apically from the crest. In addition, no standard methods for histomorphometric measurements were considered, which also contributed to histological outcome variability.

Data variability was also present at the single-study level, highlighting how bone regeneration and dimensional resorption are multifactorial processes. That is, histomorphometric and dimensional outcomes are expected to be influenced by a number of variables that might act as confounders when investigating if the two types of xenografts have any differential effects when used for ARP.

Among such confounders, the time from surgery when dimensional and histomorphometric assessment are performed might play a pivotal role. In fact, differences in the bone-formation rate might be more evident and statistically significant if clinical evaluations are performed at earlier rather than later timepoints. This hypothesis is supported by the retrospective clinical study by Di Stefano and collaborators [100], demonstrating that when CBX was used for sinus augmentation, no significant differences in NFB and residual graft material were detected between samples evaluated at different times from grafting (i.e., 3–5 months, 6–8 months, 9–12 months). These data suggest that new bone formation with CBXs occurred soon after the grafting surgery. Remarkably, early bone deposition is consistent with the significant difference detected in the amount of NFB provided by CBXs rather than ABXs in studies of ARP and sinus augmentation [38, 42]. In this regard, the clinical trials included in this systematic review also showed certain variability for the time of analysis, suggesting that the influence of this confounding factor on detecting significant differences among experimental groups remains to be clarified with appropriate studies.

Concerning the amounts of NFB that might be achieved with the two types of xenografts, one might speculate that there is an upper limit. Indeed, recent evidence showed that post-natal intramembranous bone regeneration mirrors the intramembranous ossification that occurs during embryonic bone development, with several molecular and cellular actors involved in both scenarios [110]. Because of this, the upper limit to NFB might be equal to the physiological amount of bone that patient has at the position of the arch where regeneration will occur. This might be a reasonable assumption, at least when osteoconductive grafts are used and one does not use recombinant growth factors or other drugs capable of altering bone metabolism in a relevant way. If this is the case, another factor affecting the dimensional and histomorphometric outcomes of ARP might be the position within the two arches. Indeed, a recent retrospective assessment of 6060 bone density measurements performed in 2048 patients across the two arches showed that bone density (i.e., the amount of bone by volume unit) at each position within the upper or the lower jaw exhibits significant interindividual variation, and the same patient may display significantly different densities at various positions [111]. Thus, the amount of bone growth expected should vary according to the location of the grafted site.

Finally, within the limits of the present systematic review, it is worth pointing out that the addition of a collagen carrier to ABXs did not improve dimensional and histomorphometric results compared to ABXs alone, remaining merely a technical option that allows easier biomaterial handling and application.

Thus, although the trends described in the present study suggest that ABXs and CBXs may provide different dimensional and histomorphometric outcomes when used for ARP, whether they actually do remain an open question. Answering it will require appropriate RCTs with adequate sample sizes and an experimental design carefully conceived to eliminate or at least limit the effects of several confounding factors. Possibly, studies should focus on more homogeneous patient subgroups as far as bone density is concerned (as opposed to the general population who might be subjected to ARP). Researchers should also compare xenografts grafted in symmetric or adjacent positions within the same jaw; biopsies for histomorphometric assessment should be taken soon after procedures to detect if bone formation kinetics vary between the two types of xenografts. Furthermore, the effect of carriers should be carefully investigated. While collagen added to ABXs does not seem to provide any advantage, except for better handling, it (and other carriers) might still act as a confounder, so in our opinion, studies should first compare xenografts (i.e., bone granules) with no carrier added. Finally, should any difference in histomorphometric outcomes ever be observed between ABXs and CBXs when used in ARP, future studies should investigate if this correlates with dimensional preservation of the ridge, as this point still seems unclear. Well-designed studies comparing ABXs and CBXs for ARP procedures may also allow to minimize data variability and study heterogeneity; those of data collected and discussed in the present review were indeed too high to perform any meaningful statistical analysis. This is an important limitation of the present work.

### Overall conclusions and future perspectives

The comparison between anorganic bone substitutes and CBXs for ARP procedures may provide useful information to help guide the selection of socket grafting material, but clinical data remain scant and inconclusive. Reviewed trials on ABXs and CBXs showed considerable data variation for both dimensional and histomorphometric measures of ridge preservation, which may be explained by either the intrinsic biological variability in human healing or the presence of extrinsic factors that influence the regenerative process. Overall, this systematic review supports the clinical efficacy of ARP procedures based on ABXs and CBXs, but we were unable to reach conclusions about the superiority of one xenograft over the other based on currently available data about ridge dimensional changes and histomorphometric measures. Appropriately designed clinical studies need to be carried out to directly compare anorganic bone substitutes and CBXs to assess which biomaterial provides better ridge preservation. Additionally, there is a lack of specific studies into the possible correlation between dimensional ridge preservation and histological outcomes in terms of new bone formation; such work would provide novel insights about the clinical efficacy of ARP procedures. Better characterization of these bone xenografts will be useful to guide clinical decision-making for post-extraction socket treatment and provide new perspectives on the use of different xenogenic bone substitutes.

## Data Availability

Not applicable.

## Abbreviations

ARP: Alveolar ridge preservation
ABXs: Anorganic bone xenografts
CBXs: Collagen-preserving bone xenografts
CPB: Cancellous porcine bone
EDEB: Enzyme-deantigenic equine bone
ABX: Anorganic bone xenograft
PICOT: Patient, intervention, comparison, outcome, time
RCTs: Randomized controlled trials
NFB: Newly formed bone
CaS: Calcium sulphate
DFDBA: Demineralized freeze-dried cortical bone allograft
d-PTFE: Dense polytetrafluoroethylene
EMD: Enamel matrix derivative
HA: Hydroxyapatite
ITT: Intention-to-treat
Mg/HA: Magnesium enriched-hydroxyapatite
NCHA: Nanocrystalline hydroxyapatite
PEG: Polyethylene glycol
PP: Per protocol
SBC: Straumann bone ceramics®
rhBMP-2/hydroxyapatite: Morphogenetic protein-2/HA
BCS/HA: Biphasic calcium sulphate/hydroxyapatite
β-TCP: β-Tricalcium phosphate
NFB: Newly formed bone
